# Enhanced Educational Optimization Algorithm Based on Student Psychology for Global Optimization Problems and Real Problems

**DOI:** 10.3390/biomimetics11010070

**Published:** 2026-01-14

**Authors:** Wenyu Miao, Katherine Lin Shu, Xiao Yang

**Affiliations:** 1 School of Humanities and Social Sciences, Xi’an Jiaotong University, Xi’an 710000, China; vmenu0514@stu.xjtu.edu.cn; 2 Faculty of Biology, Medicine and Health, University of Manchester, Manchester M13 9PL, UK; katherine.shu@student.manchester.ac.uk; 3 Department of Biosystems & Agricultural Engineering, College of Engineering, Michigan State University, 524 s Shaw ln, East Lansing, MI 48824, USA

**Keywords:** student psychology-based optimization algorithm, drone trajectory planning, path optimization, swarm intelligence optimization algorithm

## Abstract

To address the insufficient exploration ability, susceptibility to local optima, and limited convergence accuracy of the standard Student Psychology-Based Optimization (SPBO) algorithm in three-dimensional UAV trajectory planning, we propose an enhanced variant, Enhanced SPBO (ESPBO). ESPBO augments SPBO with three complementary strategies: (i) Time-Adaptive Scheduling, which uses normalized time (τ=t/T) to schedule global step-size shrinking, Gaussian fine-tuning, and Lévy flight intensity, enabling strong early exploration and fine late-stage exploitation; (ii) Mentor Pool Guidance, which selects a top-K mentor set and applies time-varying guidance weights to reduce misleading attraction and improve directional stability; and (iii) Directional Jump Exploration, which couples a differential vector with Lévy flights to strengthen basin-crossing while keeping the differential step bounded for robustness. Numerical experiments on CEC2017, CEC2020 and CEC2022 benchmark functions compare ESPBO with Grey Wolf Optimization (GWO), Whale Optimization Algorithm (WOA), Improved multi-strategy adaptive Grey Wolf Optimization (IAGWO), Dung Beetle Optimization (DBO), Snake Optimization (SO), Rime Optimization (RIME), and the original SPBO. We evaluate best path length, mean trajectory length, standard deviation, and convergence curves and assess statistical stability via Wilcoxon rank-sum tests (*p* = 0.05) and the Friedman test. ESPBO significantly outperforms the comparison algorithms in path-planning accuracy and convergence stability, ranking first on both test suites. Applied to 3D UAV trajectory planning in mountainous terrain with no-fly zones, ESPBO achieves an optimal path length of 199.8874 m, an average path length of 205.8179 m, and a standard deviation of 5.3440, surpassing all baselines; notably, ESPBO’s average path length is even lower than the optimal path length of other algorithms. These results demonstrate that ESPBO provides an efficient and robust solution for UAV trajectory optimization in intricate environments and extends the application of swarm intelligence algorithms in autonomous navigation.

## 1. Introduction

Path planning is a fundamental and crucial task in intelligent systems, especially in fields such as robotics, autonomous driving, and unmanned aerial vehicles (UAVs), where the effectiveness of path planning directly affects the performance and safety of the system [1]. The goal of path planning is to find an optimal or feasible path from the starting point to the target for robots, automated devices, or autonomous vehicles in complex, dynamic environments, avoiding obstacles and meeting specific constraints. With the continuous development of intelligent technologies, traditional rule-based path planning methods have gradually revealed their limitations in large-scale, complex environments [2]. To better address this issue, in recent years, path planning methods based on intelligent optimization algorithms have become a research hotspot.

Common path planning optimization methods include graph search methods, mathematical programming methods, and heuristic algorithms. Graph search methods, such as the A-star algorithm [3] and Dijkstra’s algorithm [4]. These methods are typically used for path planning in static environments, but their efficiency is relatively low in dynamic environments [5]. Mathematical programming methods solve complex mathematical models through optimization theory to obtain the optimal path. However, these methods typically require high computational costs and are difficult to handle real-time dynamic problems [6]. Therefore, intelligent optimization algorithms, especially those based on swarm intelligence and adaptive learning, have gradually become the mainstream choice for solving complex path planning problems. These algorithms, by simulating the intelligent behaviors of natural groups, such as ant foraging, bird flocking, and others, can effectively perform global optimization in more complex environments [7]. For example, the Ant Colony Optimization (ACO) algorithm [8] has long been used in classical combinatorial optimization problems, such as the Traveling Salesman Problem (TSP) [9] achieving significant results. This algorithm simulates the foraging process of ants, relying on the propagation and updating of pheromones to guide the search path, thereby continuously approaching the optimal solution. In path planning problems, ACO has also demonstrated strong potential, particularly in handling dynamic and uncertain environments, where it can adjust paths in real-time to avoid obstacles and quickly find feasible routes.

In addition to traditional swarm intelligence algorithms, many new optimization algorithms and strategies have been proposed in recent years to address path planning problems in complex dynamic environments [10]. Meanwhile, new nature-inspired optimizers explicitly target the convergence–diversity dilemma in high-dimensional search. For example, the Polar Fox Optimization (PFA) algorithm [11] models cooperative hunting behavior and alternates diversification and intensification phases to maintain population diversity while accelerating convergence; evaluated on expanded/shifted/rotated classical functions and the IEEE CEC2021 benchmark suite, PFA reports competitive performance and strong ability to escape local optima, which is directly relevant for large-scale, dynamic path planning. These advances are complemented by hybrid methodologies that combine global search with adaptive local refinement or algorithm selection to balance exploration and exploitation in large spaces—an idea already reflected in the IWOA, the Deep Q-learning-based scheduler, and the MF-DMOLSO discussed below. For example, the Improved Whale Optimization Algorithm (IWOA) [12] addresses obstacle avoidance and terrain constraints in UAV formation trajectory planning. By introducing a reverse learning mechanism to enhance the diversity of initial exploration, it improves search efficiency, particularly demonstrating better performance in dynamic and complex environments [13]. The algorithm dynamically adjusts the convergence factor and endpoint neighborhood perturbations, enabling it to quickly escape local optima and ensure better trajectory planning results. In addition, a swarm intelligence scheduling algorithm based on Deep Q-learning [14] has also been proposed in recent years, applied to multi-UAV formation trajectory planning. This algorithm uses Deep Q-learning for algorithm scheduling, selecting the most suitable optimization algorithm based on changes in the environment, thereby improving the real-time performance and adaptability of path planning. This method, by combining multiple optimization algorithms, not only improves the stability of the algorithm but also effectively handles dynamic changes in complex environments. The Multi-Objective Algorithm based on Lion Swarm Optimization (MF-DMOLSO) employs a hybrid strategy to solve multi-objective path planning problems, such as trajectory length, energy consumption, and other constrained objectives. This algorithm is particularly suitable for handling high-dimensional, dynamic path planning problems and has demonstrated excellent performance in robot and vehicle path planning. By incorporating multiple optimization strategies during the search process, MF-DMOLSO can better handle objective conflicts, improving planning accuracy and convergence speed [15]. Beyond the above, recent nature-inspired optimizers have focused explicitly on the convergence–diversity dilemma in high-dimensional search by hybridizing complementary operators and introducing adaptive controls. For example, the Crested Porcupine Optimizer (CPO) [16] maps four defensive behaviors (sight, sound, odor, physical attack) to exploration–exploitation phases and incorporates a cyclic population-reduction mechanism so that only “threatened” agents activate high-intensity updates, thereby improving both convergence rate and population diversity. In a related vein, the Adaptive Gazelle Optimization Algorithm (AGOA) [7] demonstrates a potent strategy for high-dimensional optimization. It effectively couples logistic-chaos initialization to ensure broad early coverage of the search space with adaptive Brownian motion and Lévy flights, which enable iterative step-size self-tuning. Furthermore, it incorporates a smoothed predator-effect schedule to effectively suppress late-stage oscillations. This synergistic combination of global diversification (via chaos and Lévy flights) and time-aware local refinement is a key factor contributing to its reported superior performance in both large-scale optimization accuracy and algorithmic robustness.

In Table 1, we have summarized the relevant work, covering the core operators/strategies of each method, the mechanisms for balancing exploration-development (alleviating the convergent–diversity dilemma) in high dimensions, typical benchmarks, and their applicability in unmanned aerial vehicle (UAV)/path planning.

Among the many swarm intelligence optimization algorithms, the Student Psychology-Based Optimization (SPBO) [17] is widely applied in path planning, function optimization, engineering design, and other fields [18,19] due to its simple theoretical foundation, minimal parameter settings, and ease of implementation. The core principle of this algorithm is to mimic the psychological behavior of students in the pursuit of academic achievements, constructing a self-improvement model based on changes in psychological states. Although the algorithm has achieved good results in handling some simple optimization tasks, its performance is significantly constrained when dealing with complex path planning and multi-modal optimization problems. Firstly, SPBO lacks an adaptive mechanism for updating step sizes and exploration strategies, leading to insufficient exploration in the early stages of the algorithm and an inability to effectively cover a broad solution space. In the later stages, a large step size can result in excessive adjustments, causing oscillations that affect the final convergence accuracy. Secondly, when the algorithm’s search process encounters a local optimum, it lacks sufficient guiding strategies, causing the individual update directions to become relatively random. As a result, the entire population is prone to getting trapped in specific regions, which impacts both the convergence speed and the effective discovery of the global optimum. Finally, the “random step size” strategy of the SPBO algorithm fails to effectively address multi-modal optimization problems. Particularly in high-dimensional solution spaces, the algorithm is often trapped by local extrema and lacks the ability to traverse different solution basins, thereby affecting the algorithm’s global search efficiency.

To address the shortcomings of the SPBO algorithm in complex path planning, this paper proposes an improved algorithm based on time-adaptive scheduling, mentor pool-guided updates, and directional jumps (ESPBO). By introducing a time-adaptive scheduling strategy, the step size and exploration intensity are dynamically adjusted, achieving an adaptive balance of “strong exploration in the early stages and strong exploitation in the later stages,” therefore refining both the convergence speed and the accuracy of the algorithm. Combined with the mentor pool-guided strategy, the top K individuals with the highest fitness in the population are selected as the “mentor pool,” providing diversified guidance information to enhance the search direction and avoid getting trapped in local optima. Additionally, through the directional jump exploration strategy, differential vectors and Lévy flights are used to enhance the ability to search across basins, helping the algorithm escape local extrema more quickly in multi-modal optimization problems, thereby improving global search efficiency. The proposed strategies improve the algorithm’s ability to adapt and optimize in changing environments.

The main contributions of this paper are as follows:

(1) Proposal of the ESPBO algorithm: In response to the shortcomings of the standard SPBO, the ESPBO algorithm is developed by integrating three strategies: time-adaptive scheduling, mentor pool-guided search, and directional jump exploration, and the pseudocode is provided.
(2) Numerical experiment verification: Based on the CEC2017/2020/2022 test sets, ESPBO is compared with algorithms such as GWO, RIME, and CPO. The optimization performance is validated through ablation experiments, convergence behavior analysis, and statistical tests.
(3) Application to UAV 3D trajectory planning: ESPBO is applied to UAV trajectory planning in complex terrains. Its advantages are demonstrated through metrics such as best path length, mean trajectory length, and standard deviation.

The structure of the remaining sections in this paper is as follows: Section 2 introduces the origin, mathematical model, and search strategy of the standard Student Psychology-Based Optimization (SPBO), followed by a detailed explanation of the three enhancement strategies of the proposed Enhanced Student Psychology-Based Optimization (ESPBO). Section 3 runs experiments based on the CEC2017, CEC2020 and CEC2022 benchmark functions, with ablation experiments to analyze the individual contributions and synergistic effects of each enhancement strategy. Convergence behavior is analyzed using metrics such as convergence curves and search trajectories, and stability is verified through the Wilcoxon rank-sum test and the Friedman test to validate its optimization performance. Section 4 applies ESPBO to UAV 3D path planning, testing on complex terrains to demonstrate its advantages. Section 5 concludes the study, summarizes the advantages of ESPBO, and discusses potential future research directions.

## 2. Student Psychology-Based Optimization Algorithm and the Proposed Methodology

### 2.1. Student Psychology-Based Optimization Algorithm (SPBO)

This section primarily introduces the origin and mathematical model of the SPBO algorithm. SPBO optimizes the global objective by mimicking how students allocate their efforts across different subjects based on their interests and abilities. The core idea is that students adjust their investment in each subject according to their interests and capabilities, thereby improving their overall performance and ultimately achieving the optimal solution. The following section will provide a detailed explanation of its search strategy.

Let $X_{i}=(x_{i1},x_{i2},\ldots ,x_{idim})$ represent the i−th individual $\left(i=1,2,\ldots ,N\right)$, where dim is the dimensionality of the search space, and N is the population size. $X_{best}$ represents the global best individual. The search strategy of SPBO is based on four types of students:

1. Best Student: This is the student with the highest performance, who consistently strives to maintain their leading position. The position update formula for this student is:

(1) $$\begin{array}{c} X_{best\;new}=X_{best}+k\times rand\times \left(X_{best}-X_{j}\right) \end{array}$$

where $X_{\mathrm{best}}$ is the best student’s performance, $X_{j}$ is the performance of a randomly selected student, k is a random value that can be either 1 or 2, and rand is a random number between 0 and 1.

2. Excellent Student: This student follows the efforts of the best student and may exert more effort than the other students. The position update formula for this student is:

(2) $$\begin{array}{c} X_{new}=X_{best}+rand\times \left(X_{best}-X_{i}\right)\;or{\;X}_{new}=X_{i}+rand\times \left(X_{best}-X_{i}\right)+rand\times \left(X_{mean}-X_{i}\right) \end{array}$$

where $X_{i}$ is the performance of this student, and $X_{\mathrm{mean}}$ is the average performance of the class.

3. Average Student: This student performs at an average level across all subjects, and their effort is typically based on the class’s average performance. The update formula is:

(3) $$\begin{array}{c} X_{new}=X_{i}+rand\times \left(X_{mean}-X_{i}\right) \end{array}$$

where $X_{\mathrm{mean}}$ is the average performance of the class.

4. Randomly Improved Student: These students attempt to improve their performance through random adjustments. Their efforts are randomly adjusted within the range of subject scores. The update formula is:

(4) $$\begin{array}{c} X_{new}=X_{i}+rand\times \left(X_{max}-X_{min}\right) \end{array}$$

where $X_{\min}$ and $X_{\max}$ are the minimum and maximum scores for the subject, respectively.

#### Drawbacks of SPBO and Motivation for ESPBO

While SPBO is simple and widely applicable, several limitations emerge in 3D UAV trajectory planning and multi-modal landscapes:

- Fixed Step and Update Intensity: In the absence of an adaptive step, early exploration is insufficient and late updates may oscillate, reducing convergence accuracy in complex terrains. This motivates a time-adaptive scheduling system that controls global step-size shrinking, Gaussian fine-tuning, and Lévy flight intensity via normalized time $\tau =\frac{t}{T}$ (see Equations (5)–(7)) to achieve “strong exploration early, fine exploitation late.”
- Vulnerability to Misleading Guidance: The Best/Excellent/Average/Random update rules are primarily based on $X_{\mathrm{best}}$ and $X_{\mathrm{mean}}$. If $X_{\mathrm{best}}$ is stuck in a local basin, the population may converge prematurely, hindering progress. To stabilize the directionality, we introduce a mentor pool guidance system (top-K mentors with time-varying weights), where the new population, on average, follows a convex combination of $X(i,d),{\;M}_{d},\;X_{\mathrm{best}}(d)$ (see Equation (8) and its expectation analysis). This ensures robustness to small weight perturbations and includes an anchor-stability condition for linear convergence in the later stages.
- Limited Basin-Crossing and Stability: The “random improvement step” lacks the ability to perform long jumps and can be easily trapped by local optima. To overcome this, we combine a differential vector with Lévy flights (Directional Jump Exploration). Additionally, to avoid step explosion when $r_{1}$ and $r_{2}$ have vastly different fitness values, we introduce an adaptive stability coefficient $S(r_{1},r_{2})$ (Equation (9)) to bound the difference, scaling it with F(t) in the update (Equation (10)). Since 0<S≤1, the differential contribution remains bounded, and boundary projection ensures feasibility.

These insights directly lead to the three components of ESPBO, as discussed in Section 2.2: Time-Adaptive Scheduling (Equations (5)–(7)), Mentor Pool Guidance (Equation (8) and its stability note), and Directional Jump Exploration (Equations (9) and (10)). Together, these strategies enhance early diversification, late-stage precision, and robustness, while maintaining the overall O(N⋅dim) complexity, compatible with UAV path-planning constraints.

### 2.2. Proposed Enhanced Student Psychology-Based Optimization Algorithm (ESPBO)

#### 2.2.1. Time-Adaptive Scheduling

In the traditional SPBO algorithm, the step size and update intensity are fixed, leading to insufficient exploration in the early stages, while in the later stages, the large step size can cause oscillations, reducing convergence accuracy. These issues are particularly prominent when dealing with complex path planning, as the algorithm is prone to getting trapped in local optima, which also affects the convergence speed. To address this, the ESPBO algorithm introduces the “time-adaptive scheduling” strategy, using normalized time (τ=t/T, where t is the current iteration and T is the maximum number of iterations) to control the variation in step size and weight. This achieves the effect of “strong exploration in the early stages, strong exploitation in the later stages,” thereby ensuring fast convergence and precise optimization [20].

The time-adaptive scheduling strategy includes three key update channels:

1. Global Step Size Shrinking: By gradually reducing the global step size, oscillations and excessive exploitation in the later stages are avoided. The formula is expressed as:

(5) $$\begin{array}{c} shrink\left(t\right)={\left(1-\tau \right)}^{\left(2-\tau \right)} \end{array}$$

As the iterations progress, the step size gradually decreases, thereby enhancing local search in the later stages and reducing large jumps and oscillations.

2. Gaussian Fine-Tuning Intensity: In the later stages, the step size rapidly decreases to facilitate fine search and capture of local optima. The formula is as follows:

(6) $$\begin{array}{c} gaussian\_scale\left(t\right)={\left(1-\tau \right)}^{\tau } \end{array}$$

This fine-tuning effectively reduces oscillations near the optimal solution, ensuring that the algorithm converges more stably.

3. Lévy Flight Intensity: In the early stages, a strong long-distance jump capability is provided to enhance the ability to explore the solution space, while in the later stages, this capability is gradually reduced to avoid interfering with local convergence. The formula is as follows:

(7) $$\begin{array}{c} levy\_scale\left(t\right)=(1-\tau ) \end{array}$$

This strategy ensures that, in the early stages of optimization, the algorithm can broadly explore the solution space, while in the later stages, it focuses on fine search within local optimum regions [21].

Time-adaptive scheduling can effectively accelerate the convergence process in multi-modal complex functions and reduce oscillations, thereby improving the final convergence accuracy. Compared to the traditional SPBO algorithm, it offers significant advantages.

#### 2.2.2. Mentor Pool Guidance

One issue faced by the standard SPBO algorithm is that if the current best individual gets trapped in a local optimum, the population may become misled and converge to a crowded area, slowing down the convergence speed. To address this issue, ESPBO introduces the “mentor pool guidance strategy,” which guides the search direction of other individuals by selecting the top K high-quality individuals from the population (mentor pool), thereby improving the efficiency and stability of the search.

Specifically, in each iteration, the top K individuals are selected based on fitness to form the “mentor set” M. The update formula is given by:

(8) $$\begin{array}{c} X_{new}\left(i,d\right)=X\left(i,d\right)+weight\_to\_mentor(t)\cdot u1\cdot \left(M_{d}-X\left(i,d\right)\right)+\\ weight\_to\_best(t)\cdot u2\cdot \left(X_{best}\left(d\right)-X\left(i,d\right)\right)+gaussian\_scale\left(t\right)\cdot \varepsilon \cdot (X\left(i,d\right)-X_{mean}\left(d\right)) \end{array}$$

where $X_{new}\left(i,d\right)$ is the updated solution; X(i,d) is the current position of the i−th individual in the d−th dimension; $M_{d}$ is the solution in the d−th dimension of the mentor set; $X_{best}(d)$ is the value of the global best solution in the d−th dimension; $X_{mean}(d)$ is the mean value of all individuals in the current population in the d−th dimension; u1,u2 are random numbers between [0, 1]; ε is a random number drawn from the standard normal distribution; weight_to_mentor(t)≥0 and weight_to_best(t)≥0 are weights adjusted according to the time step, controlling the influence of the mentor guidance and the global best solution; $gaussian\_scale\left(t\right)\geq 0$ is the Gaussian scaling factor adjusted according to the time step, controlling the amplitude of random perturbations.

Taking the conditional expectations of u1,u2,ϵ (given the current population and mentor pool) yields:

$$E[X_{new}\left(i,d\right)]=\theta_{0}\cdot X\left(i,d\right)+\theta_{1}\cdot M_{d}+\theta_{2}\cdot X_{best}\left(d\right)$$

where $\theta_{1}=0.5\cdot weight\_to\_mentor(t)$, $\theta_{2}=0.5\cdot weight\_to\_best(t)$, and $\theta_{0}=1-0.5\cdot (weight\_to\_mentor(t)+weight\_to\_best(t))$.

Thus, when weight_to_mentor(t)+weight_to_best(t)≤2 (which satisfies the current time weight range), $E[X_{\mathrm{new}}(i,d)]$ lies within the convex hull of $\left\{X(i,d),M_{d},X_{\mathrm{best}}(d)\right\}$, maintaining convexity in the expected sense. If there are small perturbations in the weight (weight_to_mentor(t)+δweight_to_mentor(t),weight_to_best(t)+δweight_to_best(t)) and $|\delta weight\_to\_mentor(t)|+|\delta weight\_to\_best(t)|\leq \epsilon_{w}$, the margin becomes:

$$1-0.5\cdot (weight\_to\_mentor(t)+weight\_to\_best(t)+\epsilon_{w})$$

where $\epsilon_{w}$ represents a small perturbation introduced in the algorithm, typically a small value (e.g., $\epsilon_{w}\leq 0.1$), used to test the robustness of the algorithm under slight variations, as long as $weight\_to\_mentor(t)+weight\_to\_best(t)+\epsilon_{w}\leq 2$, the property holds.

In addition, let (1) Anchor Stability: When entering the convergence stage, the mentor and global optimum satisfy:

$$|M_{d}-x^{*}\left(d\right)|,|X_{\mathrm{best}}\left(d\right)-x^{*}\left(d\right)|\leq \rho \cdot |X\left(i,d\right)-x^{*}\left(d\right)|,\rho \in \left(0,\;1\right)$$

Vanishing Variance of Interaction Terms: $\mathrm{Var}[gaussian\_scale\left(t\right)\cdot \epsilon \cdot (X(i,d)-X_{\mathrm{mean}}(d))]\to 0$. Then, during the convergence stage:

$$E|X_{\mathrm{new}}(i,d)-x^{*}(d)|\leq [1-0.5\cdot (weight\_to\_mentor(t)+weight\_to\_best(t))\cdot (1-\rho )]\cdot |X(i,d)-x^{*}(d)|+o(1)$$

Thus, the convergence is linear (because 1−0.5⋅(weight_to_mentor(t)+weight_to_mentor(t))⋅(1−ρ)<1). Since the interaction terms have zero mean and vanishing variance, they do not disrupt convergence.

This strategy effectively prevents the population from being misled, enhancing the search directionality and stability. By dynamically adjusting the weights of different guidance information, ESPBO can fully utilize diverse high-quality directions in the early stages and gradually focus on the region around the global optimum in the later stages, ensuring a stable and efficient search.

#### 2.2.3. Directional Jump Exploration

The “random improvement step” strategy of the standard SPBO algorithm lacks the ability to cross basins and is prone to getting trapped in local extrema when encountering complex multi-modal functions. To address this issue, ESPBO introduces the “Directional Jump Exploration Strategy,” which enhances the ability to cross basins through differential vectors and Lévy flights, ensuring that the algorithm can jump into low-value basins in the early stages and increase the chances of finding the global optimum. In this strategy, the differential vector generates directional updates by selecting random individuals r1 and r2:

(9) $$\begin{array}{c} {diff}_{d}=S(r_{1},r_{2})\cdot \left(X\left(r1,d\right)-X(r2,d)\right) \end{array}$$

The adaptive stability coefficient is defined as:

$$\begin{array}{cccc} & S(r_{1},r_{2})=\frac{1}{1+\beta \cdot \Delta f},\Delta f=\frac{|f(X(r_{1}))-f(X(r_{2}))|}{|f(X(r_{1}))|+|f(X(r_{2}))|+\varepsilon } & & \end{array}$$

where β>0 controls the decay strength and ε>0 (e.g., $1\times 10^{-12}$) ensures numerical stability. Because Δf is normalized, S is invariant to linear rescaling of the fitness function. When $r_{1}$ and $r_{2}$ have similar fitness (i.e., Δf→0), S→1, and the original step is recovered. When their fitness differs significantly, S decreases, thereby damping the difference vector and improving robustness under extreme pairings.

Subsequently, using the following equation:

(10) $$\begin{array}{c} X_{new}\left(i,d\right)=X\left(i,d\right)+F(t)\cdot u\cdot {diff}_{d}+levy\_scale\left(t\right)\cdot L\cdot u^{\prime }\cdot \left(X_{best}\left(d\right)-X\left(i,d\right)\right) \end{array}$$

where F(t) is the weight adjusted according to the time step, used to control the influence of the differential vector in the position update. u and $u^{\prime }$ are random numbers in the range [0, 1]. L is a one-dimensional Lévy α-stable random variable, denoted L∼Lévy(β,σ), where β is the characteristic exponent governing the heavy-tailed jump lengths, with 1<β≤2. The tail obeys P(|L|>l)∝l^{−β} as l→∞; smaller β implies more frequent long jumps. σ is the scale parameter. Since the time factor levy_scale(t) in Equation (7) already modulates the jump magnitude, we set σ=1 and use levy_scale(t) to provide the decaying scale over time. In all experiments we set β=1.5. In Equation (10), L is a scalar step-length multiplier with direction given by $\left(X_{best}\left(d\right)-X\left(i,d\right)\right)$, $u^{\prime }\in [0,\;1]$ serves as an attenuation factor, and boundary projection is applied to keep the solution feasible.

Since $0<S(r_{1},r_{2})\leq 1$, the contribution of the differential term is bounded:

$$\begin{array}{cccc} & |F(t)\cdot u\cdot {\mathrm{diff}}_{d}|\leq F(t)\cdot |u|\cdot |X(r_{1},d)-X(r_{2},d)| & & \end{array}$$

As Δf increases, S decreases monotonically, reducing the update magnitude and preventing instability when $r_{1}$ and $r_{2}$ have strongly different fitness.

This leapfrogging exploration effectively avoids local optima and increases the chances of global optimization. With this strategy, ESPBO can more quickly escape local optima and enter potential global optimum regions when handling complex multi-modal optimization problems.

By introducing these improvement strategies, ESPBO demonstrates stronger global search capabilities and higher convergence efficiency when handling complex multi-modal optimization problems. The time-adaptive scheduling ensures sufficient exploration in the early stages and fine optimization in the later stages. The mentor pool guidance prevents the population from being misled, enhancing the stability and directionality of the search, while the directional jump exploration strengthens the ability to cross basins, avoiding the problem of local optima. The combination of these three strategies significantly enhances the adaptability of ESPBO in dynamic environments, enabling it to better address complex constraints and uncertain environmental changes, thus achieving better optimization results.

#### 2.2.4. Complexity Analysis

Let N be the population size, dim the decision dimension, T the iteration budget, K the mentor-pool size, and $C_{\mathrm{eval}}(\dim)$ the per-solution objective evaluation cost.

For baseline SPBO, each iteration updates N solutions in O(N⋅dim) arithmetic and evaluates N objectives in $O(N\cdot C_{\mathrm{eval}})$. Therefore, the total complexity is $O(T\cdot N\cdot (\dim+C_{\mathrm{eval}}))$.

For Time-Adaptive Scheduling, the scalars shrink(t), $gaussian\_scale\left(t\right)$, and $levy\_scale\left(t\right)$ are O(1) to compute and are reused across iterations. Their application is fused into the per-dimension updates, keeping the overall complexity at O(N⋅dim).

For Mentor Pool Guidance, building the mentor set M of size K costs O(NlogK) with a size K heap or O(N) expected with partial selection; computing $X_{\mathrm{mean}}$ costs O(N⋅dim); updates remain O(N⋅dim). Therefore, the complexity for this step is O(N⋅dim+NlogK) per iteration.

For Directional Jump Exploration, forming the differential vector and applying F(t), $S(r_{1},r_{2})$, L, u, and $u^{\prime }$ is O(dim) per individual; S uses stored fitness values, which is O(1). Boundary projection is O(dim). Hence, the complexity for this step is O(N⋅dim) per iteration.

Thus, the overall ESPBO complexity is $O(T\cdot [N\cdot \dim+N\cdot C_{\mathrm{eval}}+NlogK])$ per run. With partial selection, the complexity reduces to $O(T\cdot [N\cdot \dim+C_{\mathrm{eval}}])$. For our UAV cost where $C_{\mathrm{eval}}(\dim)=O(\dim)$, both SPBO and ESPBO have O(T⋅N⋅dim) complexity.

The pseudocode of ESPBO is shown in Algorithm 1.
**Algorithm 1.** SPBO Algorithm Pseudocode.*1: Input N, dim, T, LB, UB, fobj**2: Expand bounds to 1 × dim; if LB(d) > UB(d) swap; if LB(d) == UB(d) set UB(d) += 1 × 10^−12^**3: X ← chaotic_init(N,dim,LB,UB); for i = 1…N: fit(i) = fobj(X(i,:)); [Best,idx] = min(fit); BestVec = X(idx,:)**4: for t = 1…T**5:      τ = t/T; shrink = (1 − τ)^(2 − τ); g = (1 − τ)^τ; l = (1 − τ); p = 0.34×(1 − 0.5τ); wM = 0.9(1 − τ) + 0.1; wB = 0.8(1 − τ^2^); wD = 1.2(1 − τ)**6:      Mentors ← top ⌈0.15N⌉ by fit; par = X; par1 = X**7:      for d = 1…dim**8:          RB = g·randn(N,1); RL = l·levy(N, 1, 1.5); r1,r2 ← distinct indices per i**9:          for i = 1…N**10:             xi = par(i,d); mk = Mentors(randi, k).d**11:             if fit(i) == Best**12:                 par1(i,d) = BestVec(d) + shrink·(RB(i)·(BestVec(d) − xi) + RL(i)·(mk − BestVec(d)))**13:             elseif rand < 0.5**14:                 par1(i,d) = xi + wM·rand·(mk − xi) + wB·rand·(BestVec(d) − xi) + RB(i)·randn·(xi − mean(X(:,d)))**15:             else**16:                 diff = par(r1(i),d) − par(r2(i),d); par1(i,d) = xi + wD·rand·diff + RL(i)·rand·(BestVec(d) − xi)**17:             end**18:         end**19:         U = (rand(N,1) < p); if any(U): par1(U,d) += shrink·(randU(LB(d),UB(d),|U|) − par1(U,d))*20:         else: k = p * (1-rand) + rand; par1(:,d) += k·(par(randperm(N),d) − par(randperm(N),d))*21:         par1(:,d) = clip(par1(:,d),LB(d),UB(d))**22:         for i = 1…N**23:             Xcand = X(i,:); Xcand(d) = par1(i,d); fnew = fobj(Xcand); if fnew < fit(i): X(i,:) = Xcand; fit(i) = fnew**24:         end**25:         [Best,idx] = min(fit); BestVec = X(idx,:); par = X; par1 = X**26:     end**27:     K = max(1,round(0.05N)); worst = worst_indices(fit,K); rad = 0.1(1 − τ)·(UB − LB)**28:     for i ∈ worst: cand = clip(BestVec + rad·randn(1,dim),LB,UB); f = fobj(cand); if f < fit(i): X(i,:) = cand; fit(i) = f; if f < Best: Best = f; BestVec = cand**29:     Convergence_curve(t) = Best**30: end; return Best, BestVec, Convergence_curve*

## 3. Numerical Experiments

### 3.1. Algorithm Parameter Settings

Benchmark test sets such as CEC2017 [22], CEC2020 [23] and CEC2022 [24] are widely adopted in the evolutionary computation community to provide a standardized and rigorous testbed for evaluating optimization algorithms [25]. These test sets consist of diverse function types (unimodal, multimodal, hybrid, and composition) that mimic real-world optimization challenges, including non-separability, ill-conditioning, and high-dimensional rugged landscapes [26]. By testing on these established benchmarks, researchers can objectively compare algorithm performance across a common platform, ensuring reproducibility and fairness [27]. The use of CEC suites also facilitates cross-study comparisons and meta-analyses, which are essential for advancing the field [28].

To assess the performance of ESPBO, we compare it with nine state-of-the-art algorithms on the CEC2017, CEC2020 and CEC2022 benchmark functions. These algorithms are classified into two groups: (1) traditional and widely recognized algorithms: GWO [29], WOA [30], SO [31]; (2) emerging and high-performance algorithms: COA [32], CPO [16], RIME [33], IAGWO [34] and DBO [35]. We first perform a comparative validation using the CEC-2017 test functions. The specific parameter settings for the comparison algorithms are shown in Table 2. All algorithms are independently run 30 times, and the experimental results will be presented in the subsequent sections. For each test function and its corresponding dimension, the best result will be highlighted in bold.

### 3.2. Ablation Experiment Analysis

**Figure 1:**
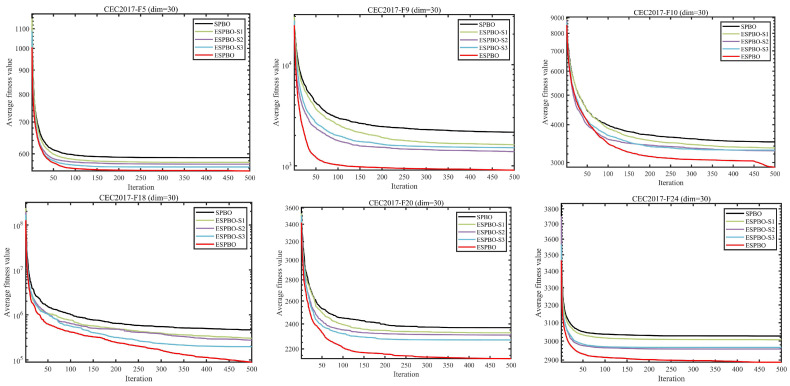
Comparison of different improvement strategies.

To analyze the separate contributions and collaborative impact of the three enhancement strategies—Time-Adaptive Scheduling (S1), Mentor Pool Guidance (S2), and Directional Jump Exploration (S3)—we conducted ablation experiments on the CEC2017 benchmark suite (dimension dim=30). Five variants of the algorithm were developed for comparison: SPBO (baseline), ESPBO S1 (containing only S1), ESPBO S2 (containing only S2), ESPBO S3 (containing only S3), and ESPBO, which incorporates all three strategies. The experimental results are shown in Figure 1.

Figure 1 presents a comparison of the iteration convergence curves of the standard SPBO algorithm and its variant strategies (ESPBO-S1, ESPBO-S2, ESPBO-S3) with ESPBO on the CEC2017 benchmark functions (dim = 30). From the convergence processes of typical functions such as CEC2017-F3, F9, and F11, it is apparent that the standard SPBO algorithm is prone to converging to local optima early on, attributed to a lack of sufficient exploration (as illustrated by the F3 function, the iterations begin to plateau shortly after starting and stagnate around the 1.2 × 10^5^ level), with slow convergence in the later stages. Although the single-strategy variants improve performance to some extent (e.g., ESPBO-S2 still shows a decreasing trend after 400 iterations in the F11 function compared to the standard SPBO), they still suffer from the defect of weak exploration ability in the early stages. In comparison, the ESPBO algorithm, which integrates three strategies, exhibits superior convergence performance across all benchmark functions. For function F3, its fitness value decreases rapidly with iterations and maintains a notable declining trend even after 500 iterations, significantly lower than other algorithms. On complex multimodal functions such as F9 and F11, ESPBO effectively avoids “positional oscillation” and sustains steady precision improvement in later iterations, validating the synergistic effects of its time-adaptive scheduling, mentor pool guidance, and directional jump mechanisms. This work establishes a performance foundation for future complex optimization tasks and trajectory planning applications.

### 3.3. Parameter Sensitivity Analysis

#### 3.3.1. Parameter Sensitivity of k in the ESPBO Component

In the original SPBO algorithm, the parameter k∈{1,2} represents two discrete learning–effort levels of the best student (regular vs. reinforced learning).

ESPBO inherits this mechanism in its SPBO-based update component to preserve the behavioral consistency of the model.

To verify whether the fixed range k∈{1,2} affects the performance of ESPBO, we conducted a sensitivity study by generalizing the update rule to

$$k∼\mathrm{Uniform}(\{1,\ldots ,K_{max}\}),$$

and testing several values of $K_{max}\in \{2,\;4,\;6,\;8\}$.

The experimental results are shown in Table 3. The overall optimal values lie within a narrow range of 1136–1137.9, with no significant performance difference observed. Meanwhile, the standard deviation remains similar across all settings. These findings suggest that ESPBO exhibits good robustness to the choice of *k*, and enlarging the range does not lead to performance improvement.

**Figure 2:**
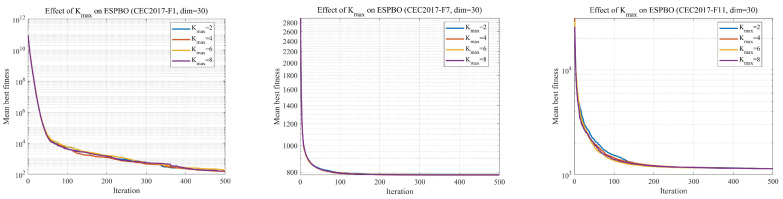
Iterative convergence curves with different $K_{max}$ values.

Figure 2 summarizes the averaged convergence curves on three representative CEC2017 functions (F1, F7, and F11) from 30 independent runs. All curves almost overlap, and the final mean best fitness values remain in a very narrow range (e.g., 1136–1137.9 on F7).

No consistent improvement is observed when enlarging $K_{max}$. On the contrary, larger $K_{max}$ occasionally leads to slightly higher variance.

These results indicate that ESPBO is highly insensitive to the choice of k, and the main performance gain of ESPBO originates from its new exploration–exploitation mechanisms rather than this SPBO component. Therefore, the original SPBO setting k∈{1,2} is retained in the final version for both semantic consistency and numerical stability.

#### 3.3.2. Mathematical Justification of the Decay Exponent

To analyze the mathematical justification of the decay exponent $\mathrm{shrink}(t)=(1-\tau {)}^{2-\tau }=(1-\tau {)}^{2-\tau }$ in Equation (5), we start by outlining the design criteria and then derive its properties and proofs. Through asymptotic expansion and scale balancing explanations, we further elucidate the behavior of this exponent.

1. Design Criteria

The decay exponent should satisfy the following design criteria (Desiderata):

1. Boundary and Monotonicity: $shrink\left(0\right)=1$, $shrink\left(T\right)=0$, and $shrink\left(t\right)$ should strictly decrease for τ∈[0,1];
2. Safe Holding: For any τ, $\left(1-\tau {)}^{2}\leq shrink\left(t\right)\leq (1-\tau \right)$;
3. Smoothness and Robustness: The function should be log-concave to ensure a “decreasing but not too fast” smooth decay;
4. Scale Balancing: The exponent should match $gaussian\_scale\left(t\right)(\tau )=(1-\tau {)}^{\tau }$ and $levy\_scale\left(t\right)(\tau )=(1-\tau )$, ensuring that the scale of fine-tuning and macro-jumps is controllable throughout the process, avoiding premature convergence or late oscillation.

2. Derivation and Properties of the Decay Exponent

To analyze the justification, we introduce the function g(τ)=(2−τ)⋅ln(1−τ), where τ=t/T is the normalized progress, and define $shrink\left(t\right)=exp(g(\tau ))$. In the implementation, $gaussian\_scale\left(t\right)(t)=(1-\tau {)}^{\tau }$ and $levy\_scale\left(t\right)(t)=(1-\tau )$.

1. Boundary and Strict Monotonicity: According to Design Criterion 1, we have $shrink\left(0\right)=1$ and $shrink\left(T\right)=0$, and $shrink\left(t\right)$ strictly decreases for τ∈(0,1). By differentiating g(τ), we show that $shrink\left(t\right)$ is decreasing throughout the interval and that the early drop is fast enough.
2. Safe Holding: Design Criterion 2 proves that for any τ∈(0,1), $\left(1-\tau {)}^{2}\leq (1-\tau {)}^{2-\tau }\leq (1-\tau \right)$. This ensures that the decay exponent is well-controlled throughout the process. The cumulative decay is bounded by $T/3\leq \int_{0}^{T}shrink(t)\mathrm{dt}\leq T/2$, and the discrete sum $\sum_{t=0}^{T}shrink(t)$ also falls within the same order of magnitude.
3. Log-Concavity: Design Criterion 3 proves that shrink(t) is log-concave. Specifically, as τ increases, the marginal decay per unit progress decreases, which helps to smooth the process during the later stages and suppress oscillations.
4. Scale Balancing: Design Criterion 4 further analyzes the relationship between the decay exponent and $gaussian\_scale\left(t\right)(\tau )$ and $levy\_scale\left(t\right)(\tau )$:

$gaussian\_scale\left(t\right)(t)\cdot shrink\left(t\right)\equiv (1-\tau {)}^{\tau }\cdot (1-\tau {)}^{2-\tau }=(1-\tau {)}^{2}$, showing that the fine-tuning scale is invariant over time;

$levy\_scale\left(t\right)(t)\cdot shrink\left(t\right)=(1-\tau )\cdot (1-\tau {)}^{2-\tau }=(1-\tau {)}^{3-\tau }$, with the exponent lying between quadratic and cubic decay, ensuring the macro-jump scale varies strictly between these two bounds.

3. Asymptotic Behavior and Slope Analysis

At the endpoints, we perform asymptotic expansion:

As τ→0, the decay exponent expands as:

$$g(\tau )=(2-\tau )ln(1-\tau )=-2\tau -\frac{1}{6}\tau^{3}+O(\tau^{4}),$$

leading to $shrink\left(\tau \right)\approx 1-2\tau +2\tau^{2}-\frac{3}{2}\tau^{3}+O(\tau^{4})$, with $shrink\prime \left(0\right)=-2$, indicating sufficient early decay.

As τ→1, let $u=1-\tau \to 0^{+}$, and we have $shrink\left(\tau \right)=u^{1+u}=u+u^{2}lnu+o(u^{2}lnu)$, so $shrink\prime \left(\tau \right)\to -1$, implying that the decay is linear during the termination phase, leading to smooth convergence without oscillation.

**Figure 3:**
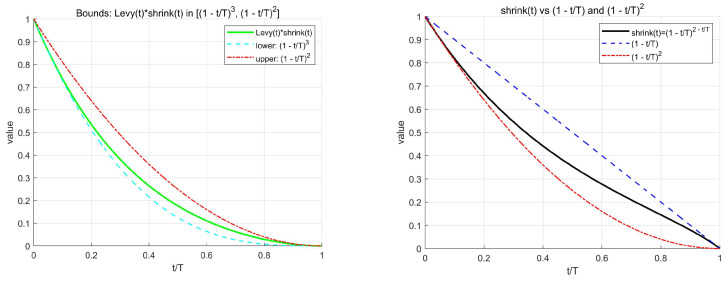
Behavior of $shrink\left(t\right)$ and its scaling relationships.

The above analysis satisfies the design criteria 1–4 and provides stronger analytical results: the combination of $shrink\left(t\right)$ with $gaussian\_scale\left(t\right)$ and $levy\_scale\left(t\right)$ forms a provable “scale balancing” throughout the entire process. Furthermore, in the natural linear exponential family, we proved the uniqueness of this choice. These results provide principled and interpretable support for selecting (2−τ) in Equation (5): fast decay in the early stages, smooth refinement in the later stages, and no additional tuning parameters required. Figure 3 displays the functional trajectory of $shrink\left(t\right)$ and its product with $gaussian\_scale\left(t\right)$ and $levy\_scale\left(t\right)$, further verifying the theoretical results.

### 3.4. Exploration and Exploitation Behavior Analysis

In metaheuristic algorithm design, the balance between exploration and exploitation is a fundamental issue. The exploration phase aims to expand the search scope by scanning unknown regions to identify potential high-quality solutions, while the exploitation phase focuses on the intensive refinement of the neighborhood of known high-quality solutions to further improve their quality [36]. This dynamic balance directly determines algorithmic performance: adequate exploration helps maintain population diversity and prevents premature convergence, whereas effective exploitation accelerates local refinement and enhances convergence precision. Such a balance enables the algorithm to not only rapidly approach the global optimum but also adapt to problems of different complexity levels, thereby demonstrating stronger adaptability and robustness [37].

To quantitatively analyze this balance, this study employs Equations (11) and (12) to calculate the proportions of exploration and exploitation, respectively. Additionally, a dimensional diversity index $Div\left(t\right)$, is introduced and computed using Equation (13), where $x_{id}$ denotes the position coordinate of the i−th individual in the d−th dimension, and ${Div}_{max}$ records the highest diversity value observed throughout the iterative process. This metric serves to monitor the dynamic characteristics of population distribution throughout the search procedure [38].

(11) $$\begin{array}{c} Exploration\left(\%\right)=\frac{Div\left(t\right)}{{Div}_{max}}\times 100 \end{array}$$

(12) $$\begin{array}{c} Exploitation\left(\%\right)=\frac{\left|Div\left(t\right)-{Div}_{max}\right|}{{Div}_{max}}\times 100 \end{array}$$

(13) $$\begin{array}{c} Div\left(t\right)=\frac{1}{D}\sum_{d=1}^{D}\frac{1}{N}\sum_{i=1}^{N}\left|median\left(x_{d}\left(t\right)\right)-x_{id}\left(t\right)\right| \end{array}$$

**Figure 4:**
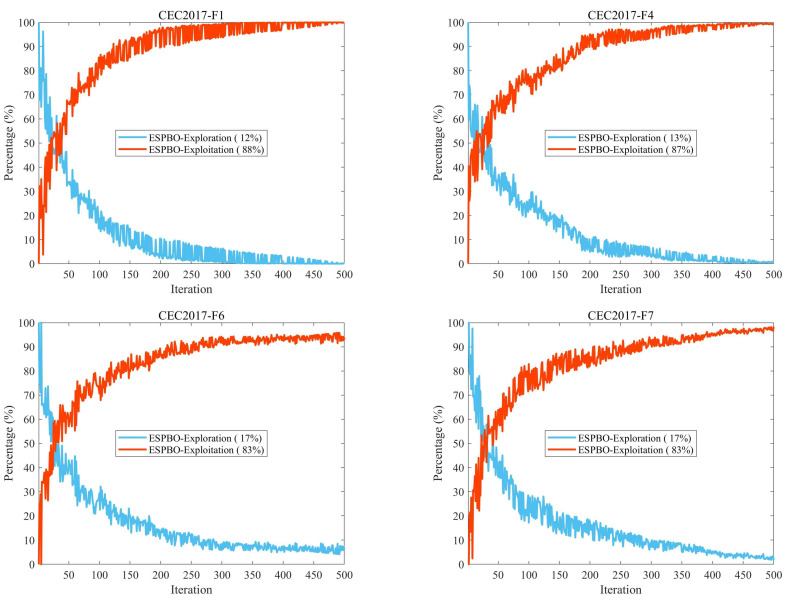

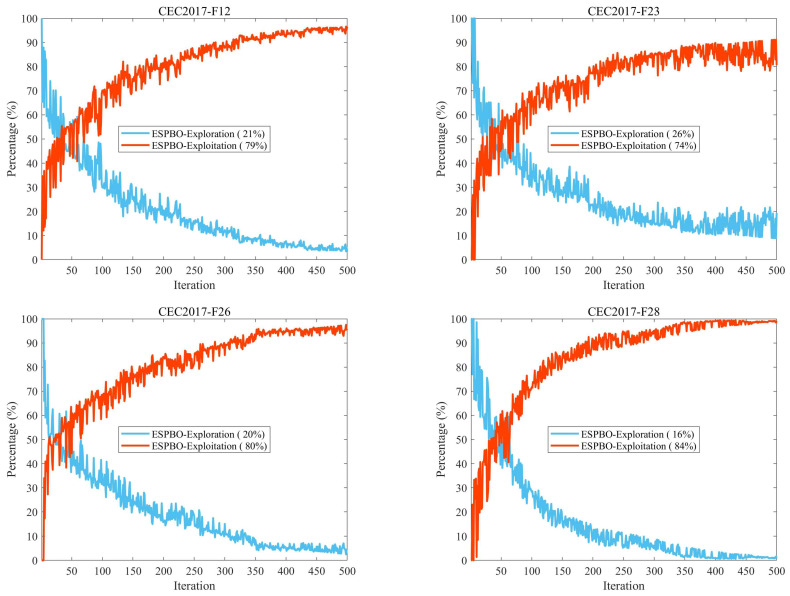
Exploration and exploitation balance ratio.

The experimental setup is shown in Figure 4.

In Equation (13), a median-centered diversity measure is employed to enhance robustness against outliers. This choice is motivated by the fact that early-stage Lévy flights and occasional restarts may produce extreme individuals. With a mean-centered diversity, such outliers can significantly distort both the center of the population and the dispersion estimate, potentially triggering incorrect “increase exploration” actions. By contrast, the use of an L1 deviation around the median (dimension-wise $|\mathrm{median}(x_{d})-x_{i,d}|$, averaged across individuals) makes the diversity measure more robust. The median is insensitive to a small number of extreme values, ensuring that a few outliers do not dominate the scheduler’s decision-making process.

The robustness of this approach stems from the median’s 50% breakdown point. When the contamination of the data is below 50%, the median remains largely unaffected by outliers, preventing any spurious increase in the spread of the population. In contrast, the mean has a 0% breakdown point and can be heavily influenced by a single extreme value, amplifying both the shift in the center and the dispersion of the population.

For populations that are symmetric and unimodal, and without notable outliers, the diversity estimates based on the median and the mean are similar. Therefore, Equation (13) does not degrade the quality of estimation for clean data, making it a consistent and effective measure in practice.

### 3.5. Convergence Behavior Analysis

**Figure 5:**
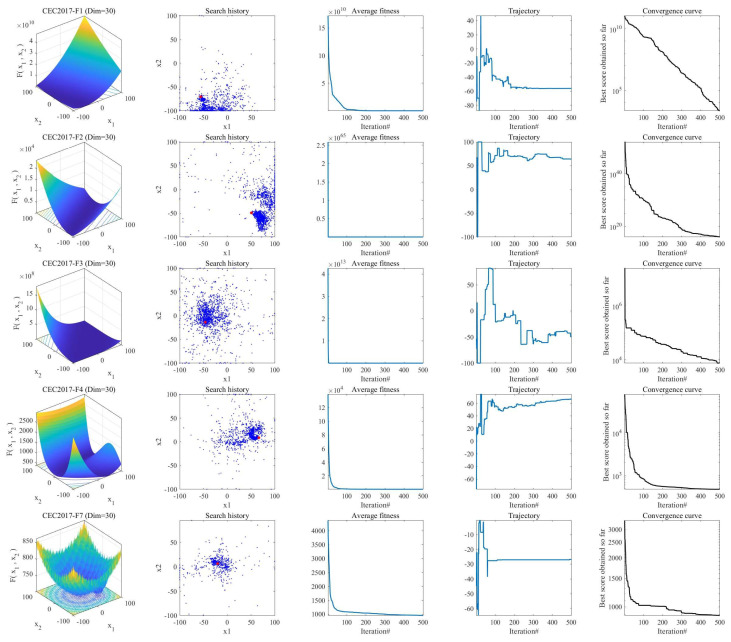

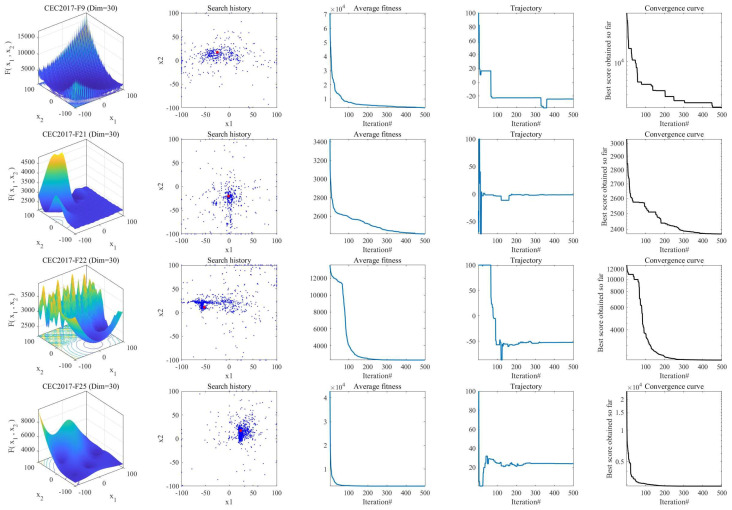
Convergence behavior of ESPBO.

For evaluating the convergence performance of the ESPBO algorithm, this study systematically analyzes its convergence behavior through multiple sets of experiments, as illustrated in Figure 5. The two-dimensional morphologies of the test functions shown in the first column intuitively reveal the complexity and challenges of the problems to be solved. In the visualization of population distribution presented in the second column, ESPBO demonstrates exceptional spatial search capability: its search agents maintain extensive coverage of the solution space while rapidly focusing on potentially optimal regions, effectively overcoming the tendency of traditional algorithms to be confined to local optima. The average fitness evolution curves in the third column reveal the convergence dynamics of the algorithm. The maintained high fitness values during early iterations reflect sufficient global exploration, while the rapid decline and subsequent stabilization of fitness values demonstrate the algorithm’s capability for smooth transition from extensive search to refined exploitation.

Of particular note, the individual trajectories shown in the fourth column transition gradually from significant initial fluctuations to stable convergence. This phenomenon indicates that ESPBO successfully resolves the oscillation issue present in the original SPBO algorithm, achieving smooth convergence. From the convergence curves, it can be observed that ESPBO performs particularly well when dealing with multimodal functions. The algorithm not only consistently escapes local optima but also accurately locates the global optimum, which benefits from its well-designed balancing mechanism. Compared to the original SPBO, ESPBO effectively addresses the issues of insufficient early-stage exploration and excessive late-stage adjustment through its dynamic regulation strategy, thereby demonstrating more stable convergence performance and higher solution accuracy in complex optimization problems.

### 3.6. Experimental Results and Analysis of CEC2017 and CEC2022 Test Suite

**Figure 6:**
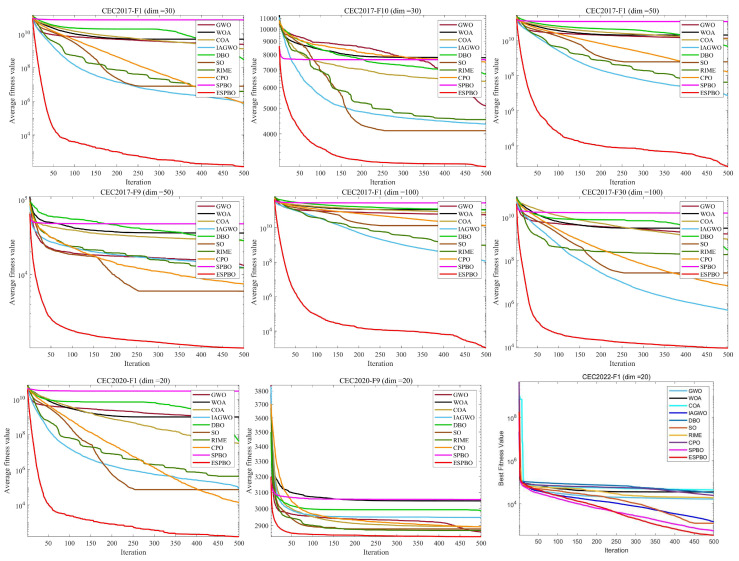
Part of the test function iterates convergence curves.

**Figure 7:**
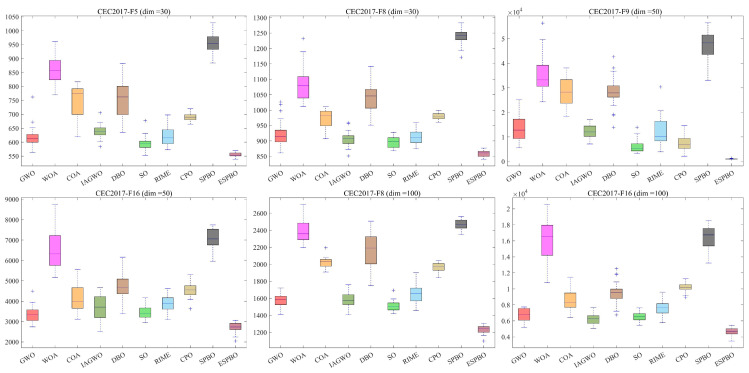
Boxplot analysis for different algorithms on the test set.

This study conducts a comparative performance analysis of ESPBO and several benchmark algorithms based on the CEC 2017, CEC 2020 and CEC 2022 standard test suites. These test suites cover four typical categories of mathematical functions: unimodal, multimodal, hybrid, and composite functions. Among them, multimodal functions, characterized by numerous local optima, effectively evaluate an algorithm’s global exploration capability. In contrast, unimodal functions contain only one global optimum and are primarily used to assess an algorithm’s local exploitation efficiency. Hybrid and composite functions, with their more complex structural features, comprehensively test the robustness of optimization algorithms in avoiding premature convergence. To ensure the fairness of the comparative experiments and mitigate the effects of randomness, for all algorithms, the population size was consistently set to 30, with the maximum number of iterations fixed at 500, and each algorithm was executed independently 30 times. Experimental results are reported in terms of the average (Ave) and standard deviation (Std), with statistically superior values highlighted in bold. The experiments were conducted on a Windows 11 platform with an Intel i5-13400 processor and 16 GB of RAM, using MATLAB 2023a as the software environment. The convergence process and solution set distribution of the algorithms are visualized through the convergence curves in Figure 6 and the box plots in Figure 7, respectively, with detailed experimental results provided in Table 4, Table 5, Table 6, Table 7 and Table 8.

Based on systematic experimental analysis of the CEC 2017 (dimensions 30/50/100) and CEC 2020/2022 (dimension 20) benchmark suites presented in Table 4, Table 5, Table 6, Table 7 and Table 8, ESPBO demonstrates significantly superior optimization performance compared to other algorithms (including GWO [29], WOA [30], COA [32], IAGWO [34], DBO [35], SO [31], RIME [33], CPO [16], and standard SPBO [17]) across various problem types (unimodal, multimodal, hybrid, and composition functions). Particularly in high-dimensional test scenarios, the advantage of ESPBO becomes more pronounced.

Based on the CEC 2022 (dim = 20) test suite, for F1 (a unimodal function), the average fitness value of ESPBO (Ave = 3.474 × 10^2^) shows a significant reduction of 46.58% compared to the standard SPBO (Ave =6.502 × 10^2^). Furthermore, its standard deviation (Std = 3.336 × 10^1^) is considerably lower than that of the standard SPBO (Std = 2.805 × 10^2^), validating the effectiveness of the time-adaptive scheduling strategy in unimodal functions, which precisely guides the search process and maintains stability. For F9 (a composition function), the Ave value of ESPBO (2.481 × 10^3^) is very close to that of the standard SPBO (Ave = 2.481 × 10^3^), and its standard deviation (Std = 2.988 × 10^−4^) is nearly zero, indicating that ESPBO consistently produces high-quality solutions without significant influence from random initial conditions and achieves rapid convergence in complex multimodal functions.

In the F1 (unimodal function) test of CEC 2017 (dim = 30), the average value of ESPBO (Ave = 1.504 × 10^2^) is significantly lower than those of other comparative algorithms, such as GWO (Ave = 3.447 × 10^9^) and WOA (Ave = 4.990 × 10^9^). Moreover, its standard deviation (Std = 1.033 × 10^2^) is notably lower than most compared algorithms, demonstrating its efficiency and stability in unimodal optimization problems. This further validates the advantage of the directional jump exploration strategy in complex problems, effectively preventing premature convergence of the population and steadily exploiting the global optimum among multiple local optima. For F7 (multimodal function), the average value of ESPBO (Ave = 7.817 × 10^2^) is 73.44% lower than that of the standard SPBO (Ave = 2.944 × 10^3^), and its standard deviation (Std = 8.944 × 10^0^) is 95.50% lower than that of the standard SPBO (Std = 1.988 × 10^2^). These results indicate that the mentor pool guidance strategy effectively avoids local optima and successfully directs the search toward the global optimum. Based on the data in Table 8, we focus on two representative functions (F1 and F7) to analyze ESPBO’s performance and its advantages over other algorithms. In the F1 (unimodal function) test of CEC 2020 (dim = 20), ESPBO shows significant superiority in unimodal optimization problems. Its average fitness value (Ave = 1.625 × 10^2^) is the lowest among all algorithms, far lower than SPBO (Ave = 2.953 × 10^10^). This performance indicates that ESPBO outperforms other algorithms in unimodal problems, particularly in minimizing path length. Moreover, the standard deviation (Std = 8.840 × 10^1^) of ESPBO is considerably lower than SPBO (Std = 4.419 × 10^9^), indicating greater stability and consistency across multiple runs. This validates the effectiveness of ESPBO’s time-adaptive scheduling strategy, which guides the search process more precisely while maintaining stability in the solutions. For the multimodal function F7, ESPBO again demonstrates its strong optimization capability. The average fitness value (Ave = 4.811 × 10^4^) for ESPBO is approximately 99.99% lower than that of SPBO (Ave = 1.802 × 10^7^), showing that ESPBO excels at finding the global optimum solution. The standard deviation (Std = 9.351 × 10^4^) for ESPBO is also much lower than SPBO (Std = 1.271 × 10^7^), further confirming its high stability and superior solution quality in multimodal problems, effectively avoiding local optima. The iterative convergence curves in Figure 6 visually demonstrate the convergence advantage of ESPBO from a dynamic process perspective. For CEC2017-F10 (dim = 30), the convergence curve of ESPBO remains consistently lower than those of other algorithms, exhibiting a steeper descent trend. By the 300th iteration, the fitness value of ESPBO has stabilized close to the optimum, while algorithms such as the standard SPBO have already become trapped in local optima. This observation clearly reflects the high efficiency of the “global exploration in early stages—local convergence in later stages” dynamic mechanism. The boxplot analysis in Figure 7 reveals the stability advantage of ESPBO from a statistical distribution perspective. Across various test scenarios, the interquartile range of ESPBO remains consistently narrow, with stable box height significantly smaller than that of the standard SPBO. The absence of outliers demonstrates ESPBO’s ability to maintain high consistency across multiple independent runs while delivering high-quality solutions.

Integrating the detailed experimental data from Table 4 to Table 7 with the visualization results in Figure 6 and Figure 7, ESPBO—through the fusion of three core strategies (time-adaptive scheduling, mentor pool guidance, and directional jump exploration)—effectively addresses the challenges faced by standard SPBO in balancing exploration and exploitation, optimizing in high-dimensional spaces, and ensuring solution stability. Across varying dimensions and diverse types of optimization tasks, ESPBO achieves comprehensive improvement in solution precision, convergence speed, and robustness, thereby providing solid experimental support for its effectiveness in practical applications such as trajectory planning problems.

### 3.7. Statistical Test

In algorithm optimization research, statistical analysis serves as a critical methodology for establishing an objective evaluation framework to compare the performance of different methods [39]. This evidence-based paradigm effectively guides researchers in selecting optimal solutions for specific problems [40]. In this study, non-parametric statistical tests, including the Wilcoxon rank-sum test and Friedman test, are employed to validate the performance of the ESPBO algorithm, while the implementation procedures and result analysis of these tests are systematically elaborated.

#### 3.7.1. Wilcoxon Rank-Sum Test

To comprehensively evaluate the overall performance of the proposed algorithm, this section introduces the Wilcoxon rank-sum test to conduct statistical analysis of the significance of differences between ESPBO and other comparative algorithms [41]. The test sets the significance level at 5%, with the null hypothesis H_0_ defined as follows: there is no statistically significant difference between the compared algorithms. If the resulting *p*-value is less than 0.05, the null hypothesis is rejected, indicating an essential difference in algorithm performance; conversely, if the *p*-value is greater than 0.05, the null hypothesis is accepted, suggesting comparable performance between algorithms. The notation “NaN” in the table indicates that no distinguishable performance difference was observed between the algorithms.

Table 9 presents the aggregated Wilcoxon rank-sum test results (α = 0.05) across all 112 benchmark functions from the CEC2022 (20-dim), CEC2020 (20-dim), and CEC2017 (30-, 50-, and 100-dim) test suites. Following the conservative convention widely adopted in leading journals, bold values in the detailed *p*-value tables in Appendix A (Table A1, Table A2, Table A3, Table A4 and Table A5) highlight cases where *p* ≥ 0.05, indicating that ESPBO exhibits no statistically significant difference from the corresponding competitor. These cases are counted as “≈” in Table 9. Even with this strict criterion, ESPBO significantly outperforms the nine state-of-the-art competitors in 924 out of 1008 pairwise comparisons (91.7%) and is never significantly inferior to any competitor.

#### 3.7.2. Friedman Mean Rank Test

To comprehensively rank the overall performance of the ESPBO algorithm, a statistical method capable of simultaneously comparing multiple related algorithms is required. The Friedman test fulfills this need precisely. As a rank-based non-parametric test, it does not rely on specific data distribution forms and is particularly suitable for evaluating the performance of multiple algorithms on the same set of benchmark functions. The test statistic [42] is determined by the following equation:

(14) $$\begin{array}{c} Q=\frac{12}{kn\left(k+1\right)}\sum_{j=1}^{k}R_{j}^{2}-3n\left(k+1\right) \end{array}$$

where n represents the number of blocks, k denotes the number of groups, $R_{j}$ indicates the sum of ranks for the j−th group. When n and k are sufficiently large, the test statistic Q approximately follows a $\chi^{2}$ distribution with k−1 degrees of freedom.

Referring to the Friedman mean rank test results outlined in Table 10, the comprehensive performance of ESPBO on the CEC 2017, CEC 2020 and CEC 2022 test suites (including dimensions 30/50/100 and 20) was evaluated and ranked against other comparative algorithms (such as GWO [29], WOA [30], COA [32], IAGWO [34], DBO [35], SO [31], RIME [33], CPO [16], and SPBO [17]). The results are presented in terms of Mean Rank (M_R) and Total Rank (T_R). As a non-parametric statistical method, the Friedman test effectively mitigates the influence of non-normal data distribution or outliers, providing a more objective reflection of the global performance differences among the algorithms.

As evidenced by the data in the table, ESPBO achieved the best Mean Rank (M_R) and Total Rank (T_R) across all test scenarios, with statistically significant differences compared to other algorithms (the test statistic Q follows a χ^2^ distribution with degrees of freedom k − 1 = 9, *p* < 0.05). Specifically, on the CEC 2017 test set (dim = 30), the average rank of ESPBO (M_R = 1.29) was considerably lower than that of the second-ranked CPO (M_R = 3.65), and its final rank (T_R = 1) demonstrated a clear lead. Even in the high-dimensional scenario of CEC 2022 (dim = 20), the average rank of ESPBO only slightly increased to 1.31, still securing the top position, while the second-ranked SPBO had an average rank of 2.38, maintaining a substantial gap. This further highlights the advantage of ESPBO in high-dimensional spaces.

From the perspective of algorithm categories, traditional algorithms (such as GWO and WOA) exhibit average ranks concentrated in the range of 4.45–8.90, while ESPBO consistently maintains an average rank between 1.29 and 1.31. This demonstrates that ESPBO outperforms competing algorithms in overall performance, emphasizing the notable advantage of its three-strategy approach.

On the CEC2020 test set, ESPBO also demonstrated significant performance advantages, with an average ranking (M_R) of 2.5 and a total ranking (T_R) consistently ranking first. Although ESPBO’s average ranking on this test set is slightly higher than its performance in CEC2017 and CEC2022, it is still significantly better than other comparison algorithms. The average rankings of traditional algorithms such as GWO (M_R = 5.5, T_R = 6) and WOA (M_R = 8.3, T_R = 9) are still concentrated in the higher range, while the novel algorithms CPO (M_R = 3.7, T_R = 2) and RIME (M_R = 3.8, T_R = 3) in recent years perform better. However, there is still a certain gap compared with ESPBO. It is worth noting that SPBO had the highest average ranking in CEC2020 (M_R = 9.9, T_R = 10), further highlighting the effectiveness of the ESPBO strategy.

**Figure 8:**
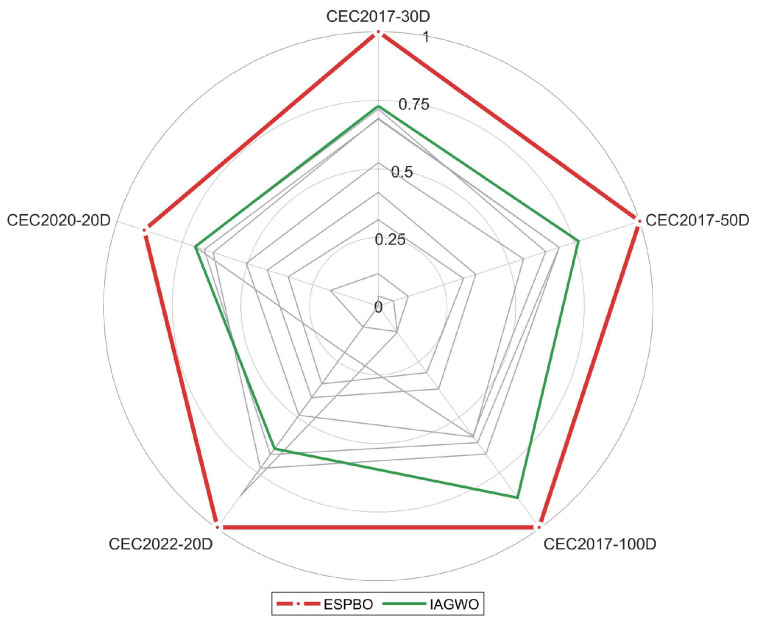
Friedman Mean Rank (radar, higher score = better).

**Figure 9:**
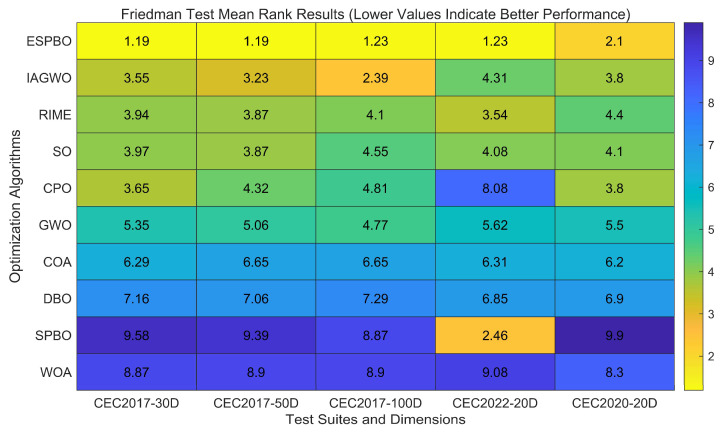
ESPBO’s Friedman test heat map.

The Friedman test results provide a comprehensive statistical comparison of the ten optimization algorithms across five benchmark scenarios with varying dimensions. As visually represented in the radar chart and heatmap, ESPBO consistently demonstrates superior performance, achieving the top ranking (Total Rank = 1) in all test suites. Figure 8 particularly highlights ESPBO’s dominant position, forming the outermost polygon that encompasses all other algorithms, indicating its comprehensive superiority across different problem types and dimensions. Figure 9 further reinforces these findings, with ESPBO showing the lowest (best) mean rank values consistently colored in the brightest shades. IAGWO emerges as the strongest competitor, securing second place in most scenarios, while traditional algorithms like WOA and SPBO generally occupy the lower performance tiers. The consistent excellence of ESPBO across diverse test conditions—from low-dimensional (20D) to high-dimensional (100D) problems—validates its robustness and adaptability, making it a highly reliable choice for complex optimization tasks including UAV trajectory planning.

Based on a comprehensive analysis of the Friedman test results in Table 10, ESPBO not only surpasses the comparison algorithms in quantitative performance metrics (such as Ave and Std) but also passes the global performance ranking test at the statistical level. Its rank distribution demonstrates “high stability and low volatility.” This advantage stems from the collaborative impact of its three core strategies: the mentor pool guidance strategy prevents the population from becoming trapped in local optima by directing the search toward promising regions, ensuring both efficiency and stability in global exploration; the time-adaptive scheduling enhances optimization capability in high-dimensional environments and improves the algorithm’s adaptability across different phases; meanwhile, the directional jump exploration strengthens global leap capability and local convergence precision, enabling rapid convergence on complex functions. Together, these strategies allow ESPBO to consistently maintain superior global performance across diverse benchmark suites and function types, providing robust statistical and visual support for its application in practical tasks such as UAV 3D trajectory planning.

## 4. Three-Dimensional UAV Path Planning

For unmanned systems, path planning is essential for mission accomplishment, particularly in UAVs, where the success of the mission heavily relies on the rationality of three-dimensional trajectory planning. Classic algorithms for path planning, such as the A-star algorithm [3], Dijkstra algorithm [4] and genetic algorithm [43], have achieved certain results in specific environments. However, their limitations become increasingly evident in complex scenarios, especially those involving intertwined factors such as mountainous terrain, high altitude, and no-fly zones [44]. Traditional methods often struggle to simultaneously satisfy multiple constraints, such as flight altitude, path length, and environmental obstacles. Consequently, they exhibit limited adaptability and efficiency when dealing with dynamic environmental changes or complex obstacles [45]. This study employs ESPBO for UAV path planning to validate its effectiveness.

**Figure 10:**
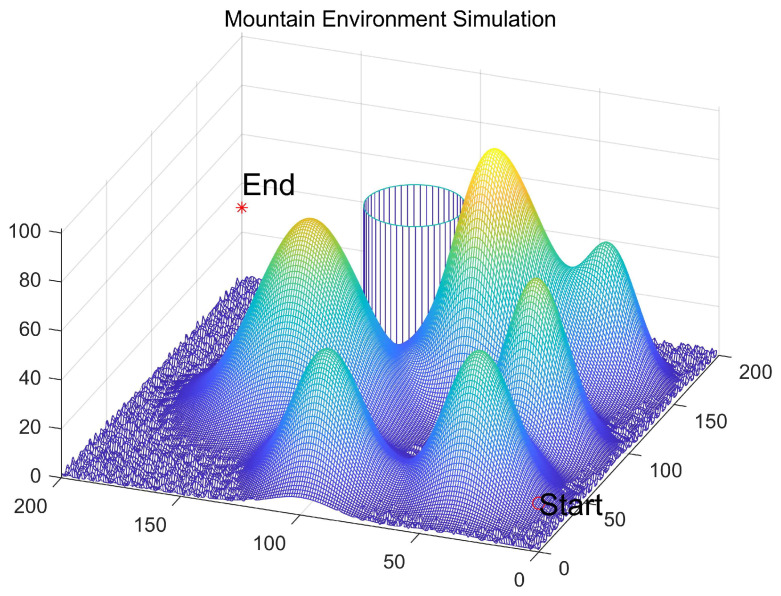
Mountain flight environment simulation.

In rugged mountainous environments, UAV flight paths face multiple complex challenges [46]. Factors such as steep mountains, abrupt weather shifts and no-fly zones [47] may pose threats to flight safety [48]. To ensure safe UAV operations, it is crucial to accurately avoid these obstacles and adverse conditions. This study addresses the UAV trajectory planning problem in mountainous terrain by incorporating topographical features, unpredictable meteorological conditions, and no-fly zones into the modeling framework, thereby forming a comprehensive path planning solution. The ground and obstacle models are illustrated in Figure 10:

(15) $$\begin{array}{c} z=\sin\left(x\right)+\sin\left(y+1\right)+\cos\left(x^{2}+y^{2}\right)+2\times \cos\left(y\right)+\sin\left(x^{2}+y^{2}\right) \end{array}$$

In UAV operations, factors like flight altitude, distance, and maximum turning angle must be controlled to maintain both safety and operational efficiency. Taking into comprehensive consideration the UAV’s maneuverability, flight path length, flight altitude, and the scale of various threat sources, this study formulates the trajectory cost function as shown in Equation (17) and conducts performance evaluation accordingly.

(16) $$\begin{array}{c} \left\{\begin{array}{c} \sum_{i=1}^{3}\omega_{i}=1\\ \omega_{i}\geq 0 \end{array}\right. \end{array}$$

To eliminate the influence of units and scales on the weighted scalarization, we first perform an ideal–nadir (utopia–nadir) normalization on the three metrics:

$$\begin{array}{c} {\hat{M}}_{i}=\frac{M_{i}-M_{i}^{\mathrm{ideal}}}{M_{i}^{\mathrm{nadir}}-M_{i}^{\mathrm{ideal}}+\varepsilon },M_{i}\in \left\{L,H,S\right\},\\ \varepsilon >0\;is\;a\;numerical\;stability\;term \end{array}$$

Here, $M_{i}$ represents metrics such as path length (L), flight altitude (H), and maximum turning angle (S), with $M_{i}^{\mathrm{ideal}}$ and $M_{i}^{\mathrm{nadir}}$ being the ideal and nadir values of these metrics, respectively. The term ε ensures numerical stability.

Based on task preferences, we define a dimensionless priority vector $p=(p_{L},p_{H},p_{S})$, where $p_{i}\geq 0$, and map it to weights as follows:

p~i=piM^-i+ϵp,ωi=p~i∑jp~j, M^-i=the mean of M^i for baseline solution or candidate set

The above formula is equivalent to “equal contribution calibration”: near the baseline solution, the marginal contributions $w_{i}\cdot {\hat{M}}_{i}$ of each metric to the objective function C are roughly balanced. When there is no particular preference, $p_{i}$ can be set equally (restoring equal weighting). The resulting weights $\omega_{i}$ satisfy the non-negativity and summation constraints in Equation (16), and are insensitive to scale changes.

Optimality and Robustness:

Pareto Optimality: When $\omega_{i}\geq 0$ and $\sum_{i}\omega_{i}=1$, the solution obtained by minimizing $C=\sum_{i}\omega_{i}{\hat{M}}_{i}$ is Pareto optimal (if a dominating solution exists, it would reduce C while maintaining or improving all other components, which would be contradictory).

Ranking Robustness (Weight Perturbation Bandwidth): Let a be the current optimal solution and b be the next best solution, with their normalized vectors ${\hat{f}}_{a},{\hat{f}}_{b}\in R_{+}^{3}$. Under the baseline weights $\omega^{0}$, we have:

$$\Delta =-(\omega^{0}{)}^{⊤}({\hat{f}}_{a}-{\hat{f}}_{b})>0$$

If $∥\omega -\omega^{0}{∥}_{1}\leq r$ and $r<\frac{\Delta }{||{\hat{f}}_{a}-{\hat{f}}_{b}|{|}_{\infty }}$, then $\omega^{⊤}{\hat{f}}_{a}<\omega^{⊤}{\hat{f}}_{b}$, meaning the ranking remains unchanged.

The choice of baseline priority p in the experiments is primarily to provide a stable reference for the task, ensuring that all optimization processes start from a unified standard, making the experimental results reliable and comparable. Using baseline priority p effectively controls the adjustment of weights for each metric, ensuring flexibility across different task scenarios while maintaining the stability and robustness of the optimization process.

(17) $$\begin{array}{c} C=\sum_{i}^{n}\left(\omega_{1}\cdot L+\omega_{2}\cdot H+\omega_{3}\cdot S+\gamma \cdot C_{\mathrm{couple}}(\hat{L},\hat{H},\hat{S})\right) \end{array}$$

where the weights $\omega_{i}$ satisfy the constraint given in Equation (16), and

γ≥0

is the coupling–strength parameter. To eliminate the influence of physical units on the cost evaluation, the path length, flight altitude, and maximum turning angle are normalized as

$$\hat{L}=\frac{L}{L_{ref}},\hat{H}=\frac{H}{H_{ref}},\hat{S}=\frac{S}{S_{ref}}$$

where $L_{ref},H_{ref},S_{ref}$ denote the reference scales of the task.

The coupling term $C_{\mathrm{couple}}(\hat{L},\hat{H},\hat{S})$ adopts a second–order multiplicative form, defined as

$$\begin{array}{cccc} & C_{\mathrm{couple}}(\hat{L},\hat{H},\hat{S})=c_{LH}\cdot \hat{L}\hat{H}+c_{LS}\cdot \hat{L}\hat{S}+c_{HS}\cdot \hat{H}\hat{S} & & \end{array}$$

where $c_{LH},c_{LS},c_{HS}\geq 0$, represent the nonlinear interaction strengths between path length and flight altitude, path length and maximum turning angle, and flight altitude and maximum turning angle, respectively.

For example, when both the path is long and the ground height deviation is large, the term $\hat{L}\cdot \hat{H}$ increases the overall cost, discouraging “high-altitude long-curve paths.” When the path is long while the curvature or second derivative is large, the term $\hat{L}\cdot \hat{S}$ suppresses undesirable trade-offs such as “increasing path length to gain smoothness.” Similarly, when both the height variation and turning intensity are high, the term $\hat{H}\cdot \hat{S}$ penalizes the compounded risk of “steep climb coupled with sharp turns.”

It is worth noting that when

γ=0,

Equation (17) degenerates to the $C=\sum_{i}^{n}\left(\omega_{1}\cdot L+\omega_{2}\cdot H+\omega_{3}\cdot S\right)$, ensuring full consistency with the experimental results already presented in this work. When stronger coupling modeling is required by the task, a positive value of γ can be selected, and the relative magnitudes of $c_{LH},c_{LS},c_{HS}$ can be adjusted according to the scenario. For instance, in environments with strict airspace or clearance safety constraints, the values of $c_{LH}$ and $c_{HS}$ may be increased accordingly.

1. Trajectory Length Constraint: This constraint limits the maximum flight distance of the UAV, aiming to ensure that the mission range remains within permissible limits. By doing so, it effectively manages onboard resources, maintains necessary endurance, and guarantees successful mission completion. The specific form of the constraint is given by Equation (18).

(18) $$\begin{array}{c} L=\sum_{i=1}^{n-1}{∥(x_{i+1},y_{i+1},z_{i+1})-(x_{i},y_{i},z_{i})∥}_{2},(i=2,3,\ldots ,n) \end{array}$$

2. Maximum Turning Angle Constraint: This constraint defines the physical limit of the UAV’s steering maneuvers, directly determining the smoothness and feasibility of the trajectory. Its mathematical model is defined by Equation (19):

(19) $$\begin{array}{c} S=\sum_{m=1}^{g}\left(cos(\varphi )-(\frac{\varphi_{i+1}\times \varphi_{i}}{\left|\varphi_{i+1}|\times |\varphi_{i}\right|}\right) \end{array}$$

To address feasibility, let $\theta_{i}$ be the turn angle between consecutive segments with $cos\;\theta_{i}=\frac{\varphi_{i+1}\cdot \varphi_{i}}{|\varphi_{i+1}||\varphi_{i}|}$. Since cosine function is strictly decreasing on [0, π], we have $\theta_{i}\leq \varphi$ if and only if $cos\;\theta_{i}\geq cos\;\varphi$, or equivalently, $cos\;\varphi -\frac{\varphi_{i+1}\cdot \varphi_{i}}{|\varphi_{i+1}||\varphi_{i}|}\leq 0$. Hence, each term in Equation (19) is strictly positive if and only if $\theta_{i}>\varphi$ and non-positive otherwise; minimizing the overall cost therefore strongly discourages violations. In our implementation, $\theta_{i}>\varphi$ is treated as a hard infeasibility (candidates are rejected or reprojected); equivalently, one can replace each term by the hinge $[cos\;\varphi -cos\;\theta_{i}{]}_{+}$ with a sufficiently large weight. Both treatments guarantee that any minimizer satisfies $\theta_{i}\leq \varphi$, thus preventing infeasible turns while preserving the cosine smoothness inside the feasible region.

3. Flight Altitude Constraint: The flight altitude is determined by safety requirements, mission specifications, and airspace regulations, all of which are critical to UAV operations. Its mathematical model is defined by Equation (20):

(20) $$\begin{array}{c} H=\sqrt{\sum_{i=1}^{n}{\left(z_{i}-\frac{1}{n}\sum_{k=1}^{g}z_{i}\right)}^{2}} \end{array}$$

where $\varphi_{i}$ represents the directional change angle between adjacent waypoints $\left(x_{i},y_{i},z_{i}\right)$ and $\left(x_{i+1},y_{i+1},z_{i+1}\right)$, and φ denotes the system-defined maximum allowable rotation angle.

To assess the performance of ESPBO in UAV 3D path planning, this study designs multiple comparative experiments. The UAV parameters are configured as follows: maximum flight altitude of 50 m, cruising speed of 20 m/s, and maximum turning angle of 60°. The testing environment is constructed as a 200 × 200 × 100 m^3^ 3D airspace, with a start coordinate of (0, 0, 20) and a target coordinate of (200, 200, 30). Under identical experimental conditions, ESPBO is compared with nine mainstream intelligent algorithms, including GWO [29], WOA [30], COA [32], GTO [49], DBO [35], SO [31], RIME [33], CPO [16], and GOA [50]. The population size for all algorithms is set to 50, with a maximum of 100 generations. To mitigate the effects of randomness, each algorithm is independently executed for 30 repeated trials.

**Figure 11:**
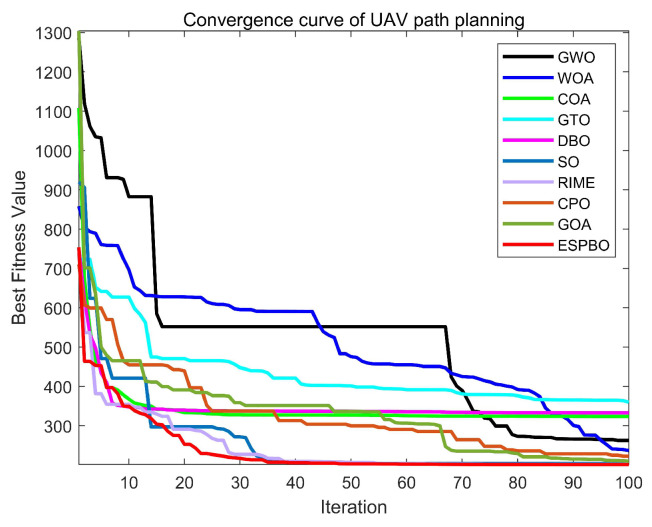
Optimal fitness search iteration curve.

Table 11 presents the statistical analysis of the experimental results, where “Best” denotes the optimal path length achieved by each algorithm, “Ave” represents the average path length over 30 independent runs, and “Std” evaluates the stability of the algorithms. The convergence curve in Figure 11 provides a dynamic perspective to complement the static data in Table 11. ESPBO’s curve exhibits two ideal phases: rapid descent and stable convergence. Its steep decline during the early iterations (approximately the first 20) demonstrates a powerful global exploration capability, enabling it to quickly locate promising regions of the solution space. In the later stages, the curve smoothly converges to its final value without significant oscillation, reflecting an effective local exploitation and refinement capability that avoids overshooting. This efficient search behavior is the direct reason for its ability to find shorter paths.

In contrast, the curves of other algorithms, such as WOA and COA, either descend slowly or plateau prematurely at higher fitness values, indicating insufficient global exploration. The slight fluctuations at the end of some curves (e.g., GTO) correlate with their larger standard deviations, revealing solution instability.

**Figure 12:**
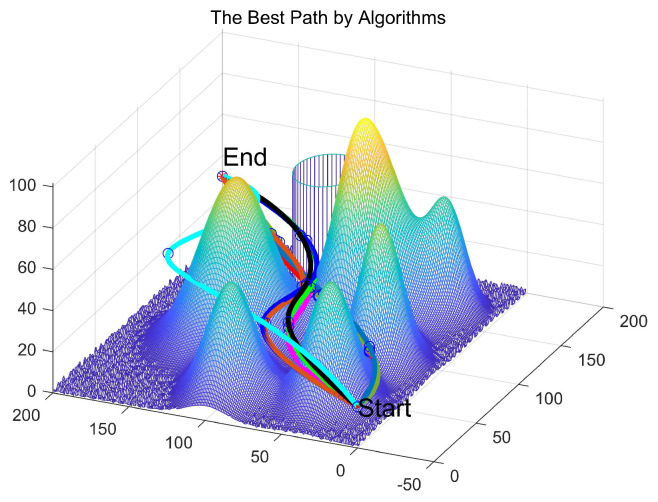
3D Path.

**Figure 13:**
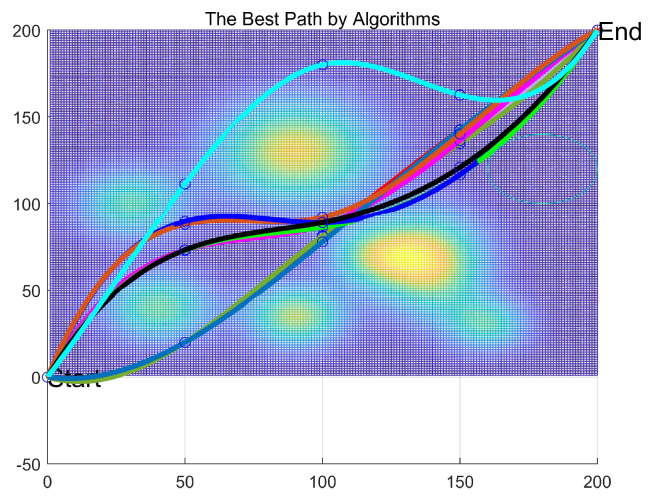
Top view of the terrain.

The 3D path visualizations in Figure 12 and Figure 13 intuitively corroborate the above analysis from an engineering standpoint. The path planned by ESPBO (recommended to be highlighted in the figure) demonstrates high practical quality: it is not only the shortest in length but also smooth, coherent, with gentle turns, and adeptly conforms to the terrain. Such a path is highly compatible with the real-world kinematic constraints of UAV flight, promoting energy efficiency and stable navigation. Conversely, paths from other algorithms show clear shortcomings: WOA’s path is lengthy and meandering; paths from some others appear “jagged” with unnecessary steep climbs and descents, which are undesirable in practical operations. Consequently, ESPBO’s superiority is evident not only in quantitative metrics but also in the practicality and quality of the paths it generates.

The statistical data presented in Table 11 is crucial for evaluating the practical potential of the algorithms. ESPBO’s lead in the Best path length (199.8874 m) demonstrates its theoretical capability to find high-quality solutions. However, its performance in Average path length (205.8179 m) and Stability (Std: 5.3440) is of greater practical significance. A particularly noteworthy finding is that ESPBO’s average path length (205.8179 m) is significantly shorter than the best path lengths discovered by all other comparative algorithms (whose best paths range from 209.4727 m to 278.8787 m). This indicates that in the vast majority of practical deployments, ESPBO can consistently output paths superior to the best-found solutions of its competitors, providing a strong justification for its deployment in real-world systems requiring high reliability.

In contrast, the shortcomings of other algorithms are apparent. For instance, while the RIME algorithm shows acceptable stability (Std = 7.1473), its inferior best and average path lengths reveal a lack of optimization power. The WOA exhibits severe flaws; its large standard deviation (97.1586) and worst path length (736.6825 m) suggest a tendency to converge on entirely infeasible solutions, rendering its performance highly unreliable. Algorithms like GTO and DBO show significant performance fluctuations (Std of 65.6775 and 50.0317, respectively), indicating sensitivity to initial conditions or random seeds and a lack of consistency. In summary, ESPBO stands out as the only algorithm that achieves top-tier performance simultaneously in both solution quality and operational stability.

Based on the comprehensive analysis above, ESPBO demonstrates significant advantages in path planning precision, algorithmic stability, and convergence performance, significantly improving the performance and robustness of UAV path optimization, especially in intricate three-dimensional environments; the path optimization capability and stability exhibited by ESPBO underscore its substantial value in practical applications.

## 5. Summary

This paper addresses two core challenges in the current field of trajectory planning optimization: insufficient solution accuracy under complex constraints, and inadequate convergence stability under multidimensional coupled constraints. Based on the Student Psychology-Based Optimization (SPBO) algorithm, this study conducts in-depth research aimed at enhancing the performance of intelligent optimization methods in complex optimization tasks and trajectory planning through algorithmic innovation. First, the paper systematically analyzes the core limitations of the standard SPBO: its fixed step-size update rule leads to an imbalance between exploration and exploitation, its reliance solely on guidance from the global best individual tends to cause premature convergence of the population, and the random step-driven local search struggles to meet the demands of high-precision optimization. To address these issues, this paper proposes an enhanced Student Psychology-Based Optimization (ESPBO) algorithm, which integrates three key strategies: time-adaptive scheduling, mentor pool guidance, and directional jump exploration. The time-adaptive scheduling strategy dynamically adjusts step sizes and exploration intensity to achieve “strong exploration in early stages and strong exploitation in later stages,” ensuring effectiveness across different phases of the search process. This approach mitigates insufficient early-stage exploration and excessive late-stage adjustments, thereby improving convergence speed and precision. The mentor pool guidance strategy selects high-fitness individuals to form a “mentor pool,” providing diversified guidance to other individuals. This enhances the directionality and stability of the search, prevents the population from being misled during the optimization process, and ensures effective discovery of the global optimum. Lastly, the directional jump exploration strategy introduces differential vectors and Lévy long jumps to strengthen the algorithm’s ability to traverse across solution basins. This helps the algorithm escape local optima in complex multimodal optimization problems and accelerates the global search process. The combination of these three strategies significantly improves the optimization stability and solution accuracy of ESPBO under complex constraints.

To validate the optimization performance of ESPBO, this study designs multiple numerical experiments based on the CEC 2017 and CEC 2022 benchmark suites, comparing it with mainstream algorithms such as GWO, COA, IAGWO, WOA, RIME, DBO, SO, CPO, and the standard SPBO. Ablation studies are further conducted to analyze the individual contributions and synergistic effects of each enhancement strategy. The experimental results demonstrate that ESPBO achieves superior performance in terms of average fitness value, convergence speed, and stability. Across the CEC 2017 (dim = 30/50/100) and CEC 2022 (dim = 20) test suites, it consistently attains the top average rank. The Wilcoxon rank-sum test reveals statistically significant differences (*p* < 0.05) in performance compared to other algorithms on most functions, while the Friedman test further confirms its overall optimization capability as the best among all compared methods. On this basis, the present study applies the ESPBO algorithm to the three-dimensional trajectory planning problem for unmanned aerial vehicles (UAVs). Using a comprehensive objective function that optimizes total trajectory length, flight altitude, and path smoothness, tests were conducted in a 3D simulation environment incorporating complex mountainous terrain and no-fly zones. Key evaluation metrics included best path length, mean trajectory length, and standard deviation. Experimental results demonstrate that ESPBO achieves superior trajectory planning outcomes compared to other algorithms, consistently excelling in both path length and stability metrics. For instance, in a typical mountainous environment, the optimal path length planned by ESPBO reached 199.8874 m, significantly outperforming other high-performance algorithms. This verifies its effectiveness in balancing path quality and planning efficiency for trajectory planning in complex environments, thereby providing a reliable solution for autonomous UAV navigation.

## 6. Future Work

The promising results of the ESPBO algorithm open up several exciting avenues for future research, aimed at enhancing its theoretical foundation, algorithmic adaptability, and practical applicability.

A primary direction involves the development of intelligent, dynamic parameter control mechanisms to transition ESPBO towards a fully self-adaptive framework. Instead of relying on static empirical values, future work will explore encoding key parameters—such as the mentor pool size, the step-size decay factor, and the Lévy flight intensity—directly into the solution representation, allowing them to evolve concurrently with the positions. Beyond this evolutionary approach, leveraging reinforcement learning to guide real-time parameter adjustment based on the search state (e.g., population diversity and convergence entropy) presents a highly promising path to eliminate manual tuning and significantly boost robustness across diverse problems.

In terms of applications, extending ESPBO to complex multi-UAV cooperative planning in dynamic, uncertain environments is a critical next step. This necessitates designing distributed or decentralized versions of the algorithm where multiple populations, each representing a UAV’s trajectory, co-evolve while managing inter-agent constraints like collision avoidance and communication maintenance. Furthermore, the algorithm’s practicality must be tested under more realistic conditions, such as the presence of moving obstacles, sudden no-fly zones, and complex meteorological disturbances like wind fields. Developing a predictive or robust optimization variant of ESPBO that can handle such uncertainties is essential for bridging the gap between simulation and real-world deployment.

Finally, while the empirical performance of ESPBO is rigorously validated, a formal theoretical analysis of its convergence properties remains an open and compelling question. Establishing a mathematical foundation using probability theory or Markov chain models to prove global convergence guarantees and to analyze convergence rates would provide deeper insights into its search dynamics and further solidify its theoretical credibility. Exploring these interconnected directions—adaptive control, complex applications, and theoretical underpinnings—will comprehensively advance the algorithm’s capabilities and scope.

## Figures and Tables

**Table 1:** Representative methods and strategies in path planning and nature-inspired optimization.

Method	Core Operators/Strategy	Convergence–Diversity Tactic	Benchmarks/Domain	Notes
A*	Graph search with heuristic	Deterministic best-first; no explicit diversity control	Grid maps; static path planning	Efficient on static maps; weak in dynamic settings
Dijkstra	Shortest path on weighted graphs	Exhaustive; no exploration–exploitation design	Grid/road networks	Optimal but costly; limited for real-time dynamics
ACO	Pheromone-guided probabilistic search	Implicit balance via evaporation/intensification	TSP; path planning	Strong adaptation to dynamic obstacles
SPBO	Student psychology-based update	Random improvement step; weak early exploration	General optimization; path planning	Baseline with simple parameters; prone to local optima
PFA	Alternating diversification/intensification	Phase cycling preserves diversity while converging	Classical (shifted/rotated), CEC2021	Competitive; good local-optima escape
IWOA	Reverse learning; adaptive convergence factor	Diversity in initialization + adaptive exploitation	UAV formation planning; dynamic terrain	Better obstacle handling; faster escape from local optima
Deep-Q scheduler	RL-based algorithm selection/scheduling	Adaptive choice maintains diversity and speed	Multi-UAV formation	Improves real-time adaptability
MF-DMOLSO	Hybrid multi-objective lion swarm framework	Joint global search + local refinement	Multi-objective path planning	Handles conflicting objectives; robust in high dim
CPO	Staged operators from porcupine defenses	Population-level adaptation; cyclic reduction	CEC2014/2017/2020/2022	Competitive vs. strong baselines
AGOA	Chaos init + adaptive Brownian/Lévy	Time-aware step-size self-tuning	CEC2017/2022	Stable late-stage behavior; robust accuracy

**Table 2:** Compare algorithm parameter settings.

Algorithms	Name of the Parameter	Value of the Parameter
GWO	α	[0, 2]
WOA	α, b, α2	[0, 2], 1, [−1, −2]
COA	temp, C	[20, 35], [0, 2]
IAGWO	$v_{rand}\left(t\right),a,\omega ,\theta$	[−20, 20], [0, 2], [0.3, 0.9], 0.5
DBO	$P_{percent}$	0.2
SO	T, c1, c2, c3, Q	0.6, 0.5, 0.05, 2, 0.25
RIME	w	5
CPO	${N,\alpha ,N}_{min},T_{f},T$	120,0.1,80,0.5,2
SPBO	NA	NA

**Table 3:** Mean and Std of optimal solutions for different $K_{\max}$.

K_max_	MeanBest	StdBest
2	1136	14.916
4	1137.2	20.114
6	1136.8	15.457
8	1137.9	20.643

**Table 4:** Experimental results of CEC2022 (dim = 20).

Function	Metric	GWO	WOA	COA	IAGWO	DBO	SO	RIME	CPO	SPBO	ESPBO
F1	Ave	1.785 × 10^6^	7.408 × 10^6^	5.805 × 10^3^	6.136 × 10^3^	5.271 × 10^5^	6.967 × 10^3^	1.471 × 10^4^	2.110 × 10^5^	2.953 × 10^10^	1.625 × 10^2^
	Std	3.961 × 10^6^	1.082 × 10^7^	3.919 × 10^3^	5.957 × 10^3^	8.784 × 10^5^	4.497 × 10^3^	7.383 × 10^3^	1.140 × 10^5^	4.419 × 10^9^	8.840 × 10^1^
F2	Ave	5.104 × 10^2^	6.291 × 10^2^	5.009 × 10^2^	7.607 × 10^2^	5.185 × 10^2^	4.603 × 10^2^	4.600 × 10^2^	5.595 × 10^2^	4.528 × 10^2^	4.468 × 10^2^
	Std	4.565 × 10^1^	7.501 × 10^1^	4.012 × 10^1^	9.449 × 10^1^	7.560 × 10^1^	1.875 × 10^1^	1.975 × 10^1^	4.624 × 10^1^	8.084 × 10^0^	1.330 × 10^1^
F3	Ave	6.071 × 10^2^	6.653 × 10^2^	6.370 × 10^2^	6.317 × 10^2^	6.330 × 10^2^	6.087 × 10^2^	6.047 × 10^2^	6.628 × 10^2^	6.047 × 10^2^	6.019 × 10^2^
	Std	4.009 × 10^0^	1.123 × 10^1^	1.889 × 10^1^	1.157 × 10^1^	1.122 × 10^1^	5.211 × 10^0^	3.130 × 10^0^	8.088 × 10^0^	1.860 × 10^0^	8.911 × 10^−1^
F4	Ave	8.557 × 10^2^	9.388 × 10^2^	8.872 × 10^2^	8.830 × 10^2^	9.268 × 10^2^	8.404 × 10^2^	8.574 × 10^2^	8.848 × 10^2^	8.638 × 10^2^	8.349 × 10^2^
	Std	2.348 × 10^1^	3.321 × 10^1^	1.059 × 10^1^	1.533 × 10^1^	2.521 × 10^1^	1.078 × 10^1^	1.895 × 10^1^	1.252 × 10^1^	1.055 × 10^1^	1.349 × 10^1^
F5	Ave	1.206 × 10^3^	3.522 × 10^3^	2.763 × 10^3^	1.810 × 10^3^	2.197 × 10^3^	1.278 × 10^3^	1.271 × 10^3^	2.975 × 10^3^	9.281 × 10^2^	9.148 × 10^2^
	Std	2.534 × 10^2^	1.065 × 10^3^	5.582 × 10^2^	3.076 × 10^2^	6.861 × 10^2^	2.397 × 10^2^	6.101 × 10^2^	3.498 × 10^2^	2.210 × 10^1^	1.101 × 10^1^
F6	Ave	1.610 × 10^4^	3.638 × 10^4^	4.381 × 10^4^	1.497 × 10^3^	3.449 × 10^4^	1.894 × 10^4^	1.704 × 10^3^	2.773 × 10^4^	6.502 × 10^2^	3.474 × 10^2^
	Std	3.881 × 10^3^	1.063 × 10^4^	1.401 × 10^4^	1.203 × 10^3^	1.097 × 10^4^	6.155 × 10^3^	7.501 × 10^2^	1.050 × 10^4^	2.805 × 10^2^	3.336 × 10^1^
F7	Ave	2.088 × 10^3^	2.237 × 10^3^	2.139 × 10^3^	2.157 × 10^3^	2.154 × 10^3^	2.080 × 10^3^	2.070 × 10^3^	2.196 × 10^3^	1.802 × 10^7^	4.811 × 10^4^
	Std	4.654 × 10^1^	6.738 × 10^1^	8.220 × 10^1^	4.157 × 10^1^	6.553 × 10^1^	2.845 × 10^1^	4.377 × 10^1^	6.985 × 10^1^	1.271 × 10^7^	9.351 × 10^4^
F8	Ave	2.261 × 10^3^	2.303 × 10^3^	2.271 × 10^3^	2.264 × 10^3^	2.332 × 10^3^	2.243 × 10^3^	2.258 × 10^3^	2.307 × 10^3^	2.231 × 10^3^	2.228 × 10^3^
	Std	5.109 × 10^1^	7.761 × 10^1^	6.133 × 10^1^	6.338 × 10^1^	7.795 × 10^1^	3.106 × 10^1^	5.082 × 10^1^	1.028 × 10^2^	1.494 × 10^0^	2.248 × 10^0^
F9	Ave	2.531 × 10^3^	2.611 × 10^3^	2.483 × 10^3^	2.482 × 10^3^	2.516 × 10^3^	2.481 × 10^3^	2.482 × 10^3^	2.560 × 10^3^	2.481 × 10^3^	2.481 × 10^3^
	Std	2.956 × 10^1^	5.277 × 10^1^	3.345 × 10^0^	6.013 × 10^0^	4.037 × 10^1^	2.324 × 10^−1^	1.033 × 10^0^	5.472 × 10^1^	2.107 × 10^−1^	2.988 × 10^−4^
F10	Ave	3.655 × 10^3^	4.927 × 10^3^	4.152 × 10^3^	3.661 × 10^3^	3.766 × 10^3^	3.018 × 10^3^	2.836 × 10^3^	4.244 × 10^3^	2.513 × 10^3^	2.750 × 10^3^
	Std	9.152 × 10^2^	1.319 × 10^3^	1.356 × 10^3^	1.295 × 10^3^	1.372 × 10^3^	4.177 × 10^2^	2.944 × 10^2^	9.260 × 10^2^	4.539 × 10^1^	4.551 × 10^2^
F11	Ave	1.610 × 10^4^	3.638 × 10^4^	4.381 × 10^4^	1.490 × 10^3^	3.449 × 10^4^	1.894 × 10^4^	1.704 × 10^3^	2.773 × 10^4^	6.502 × 10^2^	3.474 × 10^2^
	Std	3.881 × 10^3^	1.063 × 10^4^	1.401 × 10^4^	1.203 × 10^3^	1.097 × 10^4^	6.155 × 10^3^	7.501 × 10^2^	1.050 × 10^4^	2.805 × 10^2^	3.336 × 10^1^
F12	Ave	5.104 × 10^2^	6.291 × 10^2^	5.009 × 10^2^	4.607 × 10^2^	5.185 × 10^2^	4.603 × 10^2^	4.600 × 10^2^	5.595 × 10^2^	4.528 × 10^2^	4.468 × 10^2^
	Std	4.565 × 10^1^	7.501 × 10^1^	4.012 × 10^1^	2.449 × 10^1^	7.560 × 10^1^	1.875 × 10^1^	1.975 × 10^1^	4.624 × 10^1^	8.084 × 10^0^	1.330 × 10^1^

**Table 5:** Experimental results of CEC2017 (dim = 30).

Function	Metric	GWO	WOA	COA	IAGWO	DBO	SO	RIME	CPO	SPBO	ESPBO
F1	Ave	3.447 × 10^9^	4.990 × 10^9^	6.540 × 10^2^	6.451 × 10^2^	6.508 × 10^2^	6.193 × 10^2^	6.124 × 10^2^	6.020 × 10^2^	6.872 × 10^2^	1.504 × 10^2^
	Std	4.307 × 10^0^	1.032 × 10^1^	1.317 × 10^1^	1.027 × 10^1^	1.119 × 10^1^	6.406 × 10^0^	7.760 × 10^0^	7.869 × 10^−1^	5.703 × 10^0^	1.033 × 10^2^
F2	Ave	7.684 × 10^32^	1.771 × 10^35^	5.109 × 10^26^	1.131 × 10^24^	1.512 × 10^35^	2.271 × 10^27^	4.903 × 10^17^	2.195 × 10^20^	2.930 × 10^40^	1.130 × 10^5^
	Std	1.923 × 10^33^	5.066 × 10^35^	1.926 × 10^27^	3.538 × 10^24^	5.970 × 10^35^	1.112 × 10^28^	1.845 × 10^18^	5.885 × 10^20^	9.444 × 10^40^	1.442 × 10^5^
F3	Ave	6.192 × 10^4^	2.960 × 10^5^	1.151 × 10^5^	1.587 × 10^4^	9.414 × 10^4^	7.126 × 10^4^	4.864 × 10^4^	6.383 × 10^4^	2.276 × 10^5^	6.602 × 10^4^
	Std	1.414 × 10^4^	6.378 × 10^4^	3.109 × 10^4^	5.869 × 10^3^	2.334 × 10^4^	1.044 × 10^4^	1.575 × 10^4^	1.304 × 10^4^	3.918 × 10^4^	1.867 × 10^4^
F4	Ave	6.102 × 10^2^	1.202 × 10^3^	6.107 × 10^2^	8.216 × 10^2^	7.070 × 10^2^	5.612 × 10^2^	5.307 × 10^2^	5.155 × 10^2^	9.130 × 10^3^	4.299 × 10^2^
	Std	8.251 × 10^1^	2.775 × 10^2^	7.073 × 10^1^	4.676 × 10^1^	2.266 × 10^2^	3.313 × 10^1^	4.020 × 10^1^	1.266 × 10^1^	2.240 × 10^3^	2.748 × 10^1^
F5	Ave	6.228 × 10^2^	8.535 × 10^2^	7.451 × 10^2^	6.826 × 10^2^	7.587 × 10^2^	6.016 × 10^2^	6.191 × 10^2^	6.889 × 10^2^	9.588 × 10^2^	5.550 × 10^2^
	Std	3.256 × 10^1^	6.131 × 10^1^	5.316 × 10^1^	4.385 × 10^1^	5.765 × 10^1^	2.198 × 10^1^	2.954 × 10^1^	1.507 × 10^1^	2.954 × 10^1^	7.163 × 10^0^
F6	Ave	3.447 × 10^9^	4.990 × 10^9^	4.397 × 10^8^	2.195 × 10^5^	2.608 × 10^8^	6.490 × 10^6^	3.958 × 10^6^	8.318 × 10^5^	6.614 × 10^10^	1.504 × 10^2^
	Std	2.097 × 10^9^	1.799 × 10^9^	5.316 × 10^8^	2.998 × 10^5^	1.522 × 10^8^	7.595 × 10^6^	2.360 × 10^6^	8.606 × 10^5^	9.163 × 10^9^	1.033 × 10^2^
F7	Ave	9.182 × 10^2^	1.303 × 10^3^	1.178 × 10^3^	1.063 × 10^3^	1.010 × 10^3^	9.143 × 10^2^	8.707 × 10^2^	9.389 × 10^2^	2.944 × 10^3^	7.817 × 10^2^
	Std	7.030 × 10^1^	8.645 × 10^1^	1.221 × 10^2^	6.690 × 10^1^	8.708 × 10^1^	4.419 × 10^1^	3.430 × 10^1^	1.877 × 10^1^	1.988 × 10^2^	8.944 × 10^0^
F8	Ave	9.146 × 10^2^	1.066 × 10^3^	9.756 × 10^2^	9.518 × 10^2^	1.044 × 10^3^	8.970 × 10^2^	9.094 × 10^2^	9.824 × 10^2^	1.243 × 10^3^	8.623 × 10^2^
	Std	4.015 × 10^1^	5.367 × 10^1^	2.668 × 10^1^	2.741 × 10^1^	4.925 × 10^1^	1.749 × 10^1^	3.269 × 10^1^	1.753 × 10^1^	2.749 × 10^1^	1.136 × 10^1^
F9	Ave	2.590 × 10^3^	1.004 × 10^4^	8.065 × 10^3^	4.202 × 10^3^	6.365 × 10^3^	2.258 × 10^3^	3.045 × 10^3^	1.370 × 10^3^	1.997 × 10^4^	9.113 × 10^2^
	Std	7.345 × 10^2^	3.552 × 10^3^	1.551 × 10^3^	9.213 × 10^2^	2.187 × 10^3^	6.169 × 10^2^	1.325 × 10^3^	4.550 × 10^2^	2.432 × 10^3^	8.710 × 10^0^
F10	Ave	4.897 × 10^3^	7.711 × 10^3^	6.098 × 10^3^	5.898 × 10^3^	6.503 × 10^3^	4.214 × 10^3^	4.765 × 10^3^	7.513 × 10^3^	7.646 × 10^3^	2.937 × 10^3^
	Std	9.274 × 10^2^	8.140 × 10^2^	9.236 × 10^2^	8.829 × 10^2^	1.404 × 10^3^	7.445 × 10^2^	5.774 × 10^2^	4.300 × 10^2^	2.482 × 10^2^	2.602 × 10^2^
F11	Ave	3.068 × 10^3^	9.901 × 10^3^	1.809 × 10^3^	1.265 × 10^3^	2.008 × 10^3^	1.416 × 10^3^	1.364 × 10^3^	1.273 × 10^3^	2.354 × 10^4^	1.136 × 10^3^
	Std	1.660 × 10^3^	4.508 × 10^3^	4.049 × 10^2^	5.788 × 10^1^	7.128 × 10^2^	1.084 × 10^2^	6.175 × 10^1^	2.594 × 10^1^	6.215 × 10^3^	1.688 × 10^1^
F12	Ave	8.150 × 10^7^	6.372 × 10^8^	1.313 × 10^7^	1.829 × 10^6^	5.370 × 10^7^	5.161 × 10^6^	1.810 × 10^7^	1.131 × 10^6^	1.035 × 10^10^	3.649 × 10^5^
	Std	5.714 × 10^7^	4.188 × 10^8^	1.062 × 10^7^	1.330 × 10^6^	6.652 × 10^7^	5.013 × 10^6^	1.558 × 10^7^	8.247 × 10^5^	2.478 × 10^9^	2.812 × 10^5^
F13	Ave	2.846 × 10^7^	1.146 × 10^7^	1.685 × 10^5^	2.769 × 10^4^	1.029 × 10^7^	5.223 × 10^4^	1.115 × 10^5^	2.434 × 10^4^	1.374 × 10^10^	2.367 × 10^3^
	Std	1.007 × 10^8^	8.898 × 10^6^	1.636 × 10^5^	3.113 × 10^4^	1.885 × 10^7^	4.980 × 10^4^	7.232 × 10^4^	1.767 × 10^4^	3.956 × 10^9^	9.453 × 10^2^
F14	Ave	7.733 × 10^5^	2.674 × 10^6^	4.092 × 10^5^	6.031 × 10^3^	3.149 × 10^5^	6.660 × 10^4^	9.561 × 10^4^	2.017 × 10^3^	2.941 × 10^7^	2.526 × 10^4^
	Std	1.056 × 10^6^	2.799 × 10^6^	4.479 × 10^5^	6.267 × 10^3^	3.209 × 10^5^	6.429 × 10^4^	6.860 × 10^4^	4.429 × 10^2^	2.106 × 10^7^	2.422 × 10^4^
F15	Ave	7.028 × 10^5^	5.573 × 10^6^	2.330 × 10^4^	5.209 × 10^3^	1.101 × 10^5^	1.386 × 10^4^	2.071 × 10^4^	4.874 × 10^3^	4.379 × 10^9^	2.106 × 10^3^
	Std	1.407 × 10^6^	1.117 × 10^7^	1.856 × 10^4^	3.674 × 10^3^	2.054 × 10^5^	1.060 × 10^4^	1.356 × 10^4^	2.401 × 10^3^	1.509 × 10^9^	7.416 × 10^2^
F16	Ave	2.603 × 10^3^	4.252 × 10^3^	2.992 × 10^3^	2.721 × 10^3^	3.356 × 10^3^	2.584 × 10^3^	2.710 × 10^3^	3.067 × 10^3^	5.236 × 10^3^	2.060 × 10^3^
	Std	3.361 × 10^2^	5.289 × 10^2^	3.784 × 10^2^	3.406 × 10^2^	4.265 × 10^2^	2.736 × 10^2^	3.184 × 10^2^	2.149 × 10^2^	4.202 × 10^2^	1.960 × 10^2^
F17	Ave	2.022 × 10^3^	2.782 × 10^3^	2.282 × 10^3^	2.264 × 10^3^	2.680 × 10^3^	2.227 × 10^3^	2.194 × 10^3^	2.028 × 10^3^	6.697 × 10^3^	1.842 × 10^3^
	Std	1.413 × 10^2^	2.679 × 10^2^	2.451 × 10^2^	2.530 × 10^2^	2.684 × 10^2^	1.889 × 10^2^	2.014 × 10^2^	1.035 × 10^2^	4.963 × 10^3^	8.245 × 10^1^
F18	Ave	2.571 × 10^6^	1.038 × 10^7^	2.869 × 10^6^	1.297 × 10^5^	3.234 × 10^6^	1.226 × 10^6^	1.864 × 10^6^	1.502 × 10^5^	7.751 × 10^7^	1.376 × 10^5^
	Std	4.149 × 10^6^	9.806 × 10^6^	4.333 × 10^6^	9.836 × 10^4^	5.238 × 10^6^	1.197 × 10^6^	2.212 × 10^6^	9.617 × 10^4^	3.634 × 10^7^	8.455 × 10^4^
F19	Ave	1.001 × 10^6^	1.886 × 10^7^	3.105 × 10^4^	7.599 × 10^3^	6.412 × 10^6^	1.834 × 10^4^	1.072 × 10^4^	7.571 × 10^3^	5.232 × 10^9^	2.458 × 10^3^
	Std	1.326 × 10^6^	1.922 × 10^7^	5.382 × 10^4^	9.174 × 10^3^	1.409 × 10^7^	1.783 × 10^4^	9.282 × 10^3^	6.492 × 10^3^	2.066 × 10^9^	1.007 × 10^3^
F20	Ave	2.441 × 10^3^	2.940 × 10^3^	2.640 × 10^3^	2.589 × 10^3^	2.757 × 10^3^	2.576 × 10^3^	2.515 × 10^3^	2.429 × 10^3^	3.284 × 10^3^	2.156 × 10^3^
	Std	1.447 × 10^2^	1.967 × 10^2^	2.355 × 10^2^	1.683 × 10^2^	2.044 × 10^2^	1.626 × 10^2^	1.934 × 10^2^	1.337 × 10^2^	1.339 × 10^2^	8.290 × 10^1^
F21	Ave	2.397 × 10^3^	2.641 × 10^3^	2.473 × 10^3^	2.442 × 10^3^	2.544 × 10^3^	2.404 × 10^3^	2.417 × 10^3^	2.479 × 10^3^	2.718 × 10^3^	2.360 × 10^3^
	Std	1.874 × 10^1^	4.611 × 10^1^	4.937 × 10^1^	5.774 × 10^1^	4.715 × 10^1^	2.227 × 10^1^	2.582 × 10^1^	1.501 × 10^1^	3.398 × 10^1^	3.089 × 10^1^
F22	Ave	4.998 × 10^3^	8.576 × 10^3^	4.399 × 10^3^	3.095 × 10^3^	4.860 × 10^3^	4.403 × 10^3^	4.657 × 10^3^	2.311 × 10^3^	9.147 × 10^3^	4.230 × 10^3^
	Std	1.823 × 10^3^	1.518 × 10^3^	2.505 × 10^3^	1.809 × 10^3^	2.741 × 10^3^	1.840 × 10^3^	1.987 × 10^3^	4.174 × 10^0^	4.082 × 10^2^	9.301 × 10^2^
F23	Ave	2.781 × 10^3^	3.124 × 10^3^	2.864 × 10^3^	2.875 × 10^3^	2.986 × 10^3^	2.812 × 10^3^	2.791 × 10^3^	2.848 × 10^3^	3.049 × 10^3^	2.707 × 10^3^
	Std	4.544 × 10^1^	8.204 × 10^1^	7.492 × 10^1^	8.269 × 10^1^	8.340 × 10^1^	3.914 × 10^1^	3.727 × 10^1^	1.733 × 10^1^	2.898 × 10^1^	1.039 × 10^1^
F24	Ave	2.978 × 10^3^	3.260 × 10^3^	3.025 × 10^3^	3.033 × 10^3^	3.219 × 10^3^	2.955 × 10^3^	2.949 × 10^3^	3.016 × 10^3^	3.235 × 10^3^	2.911 × 10^3^
	Std	7.303 × 10^1^	8.802 × 10^1^	5.446 × 10^1^	1.030 × 10^2^	1.094 × 10^2^	4.255 × 10^1^	3.935 × 10^1^	1.714 × 10^1^	1.993 × 10^1^	2.781 × 10^1^
F25	Ave	3.014 × 10^3^	3.215 × 10^3^	2.992 × 10^3^	2.915 × 10^3^	2.970 × 10^3^	2.947 × 10^3^	2.921 × 10^3^	2.912 × 10^3^	1.040 × 10^4^	2.884 × 10^3^
	Std	4.939 × 10^1^	8.520 × 10^1^	6.402 × 10^1^	2.694 × 10^1^	6.311 × 10^1^	2.937 × 10^1^	1.982 × 10^1^	1.902 × 10^1^	1.639 × 10^3^	6.176 × 10^−1^
F26	Ave	4.886 × 10^3^	8.394 × 10^3^	6.804 × 10^3^	5.506 × 10^3^	6.868 × 10^3^	5.399 × 10^3^	5.052 × 10^3^	5.179 × 10^3^	8.539 × 10^3^	3.901 × 10^3^
	Std	4.779 × 10^2^	9.589 × 10^2^	1.479 × 10^3^	1.520 × 10^3^	1.044 × 10^3^	5.549 × 10^2^	7.089 × 10^2^	1.172 × 10^3^	2.865 × 10^2^	6.441 × 10^2^
F27	Ave	3.275 × 10^3^	3.452 × 10^3^	3.293 × 10^3^	3.288 × 10^3^	3.362 × 10^3^	3.296 × 10^3^	3.243 × 10^3^	3.276 × 10^3^	3.402 × 10^3^	3.201 × 10^3^
	Std	3.300 × 10^1^	1.362 × 10^2^	4.960 × 10^1^	5.295 × 10^1^	9.111 × 10^1^	3.050 × 10^1^	1.791 × 10^1^	9.779 × 10^0^	3.419 × 10^1^	5.321 × 10^0^
F28	Ave	3.519 × 10^3^	3.993 × 10^3^	3.391 × 10^3^	3.280 × 10^3^	3.856 × 10^3^	3.364 × 10^3^	3.311 × 10^3^	3.280 × 10^3^	7.144 × 10^3^	3.169 × 10^3^
	Std	1.599 × 10^2^	2.883 × 10^2^	6.815 × 10^1^	2.057 × 10^1^	9.862 × 10^2^	5.839 × 10^1^	5.443 × 10^1^	2.540 × 10^1^	6.656 × 10^2^	3.362 × 10^1^
F29	Ave	3.979 × 10^3^	5.372 × 10^3^	4.178 × 10^3^	4.354 × 10^3^	4.490 × 10^3^	4.065 × 10^3^	4.079 × 10^3^	4.030 × 10^3^	5.381 × 10^3^	3.430 × 10^3^
	Std	1.753 × 10^2^	5.287 × 10^2^	3.021 × 10^2^	4.031 × 10^2^	3.849 × 10^2^	2.452 × 10^2^	2.284 × 10^2^	1.153 × 10^2^	3.945 × 10^2^	8.993 × 10^1^
F30	Ave	1.767 × 10^7^	5.857 × 10^7^	8.873 × 10^5^	7.393 × 10^4^	3.112 × 10^6^	2.153 × 10^5^	6.606 × 10^5^	1.067 × 10^5^	1.558 × 10^9^	7.557 × 10^3^
	Std	1.568 × 10^7^	4.895 × 10^7^	8.496 × 10^5^	1.881 × 10^5^	5.236 × 10^6^	2.577 × 10^5^	4.923 × 10^5^	5.857 × 10^4^	5.287 × 10^8^	1.056 × 10^3^

**Table 6:** Experimental results of CEC2017 (dim = 50).

Function	Metric	GWO	WOA	COA	IAGWO	DBO	SO	RIME	CPO	SPBO	ESPBO
F1	Ave	1.077 × 10^3^	1.401 × 10^3^	1.226 × 10^3^	1.140 × 10^3^	1.306 × 10^3^	1.015 × 10^3^	1.078 × 10^3^	1.217 × 10^3^	1.565 × 10^3^	9.345 × 10^2^
	Std	4.164 × 10^1^	5.464 × 10^1^	3.559 × 10^1^	3.539 × 10^1^	9.552 × 10^1^	3.272 × 10^1^	5.680 × 10^1^	3.223 × 10^1^	3.325 × 10^1^	1.593 × 10^1^
F2	Ave	1.521 × 10^54^	1.463 × 10^80^	1.236 × 10^61^	4.165 × 10^53^	2.932 × 10^64^	6.573 × 10^56^	1.082 × 10^43^	8.192 × 10^45^	5.774 × 10^73^	1.502 × 10^12^
	Std	4.195 × 10^54^	6.018 × 10^80^	6.151 × 10^61^	2.278 × 10^54^	1.552 × 10^65^	3.128 × 10^57^	5.592 × 10^43^	3.773 × 10^46^	2.099 × 10^74^	5.090 × 10^12^
F3	Ave	1.750 × 10^5^	3.089 × 10^5^	3.260 × 10^5^	8.082 × 10^4^	2.875 × 10^5^	1.608 × 10^5^	2.179 × 10^5^	1.834 × 10^5^	3.620 × 10^5^	2.549 × 10^5^
	Std	3.607 × 10^4^	1.129 × 10^5^	7.750 × 10^4^	1.316 × 10^4^	8.861 × 10^4^	2.139 × 10^4^	4.274 × 10^4^	2.369 × 10^4^	4.785 × 10^4^	3.433 × 10^4^
F4	Ave	1.460 × 10^3^	4.866 × 10^3^	1.895 × 10^3^	7.197 × 10^2^	1.850 × 10^3^	8.637 × 10^2^	6.787 × 10^2^	7.091 × 10^2^	2.161 × 10^4^	4.385 × 10^2^
	Std	4.759 × 10^2^	1.264 × 10^3^	9.902 × 10^2^	6.290 × 10^1^	2.143 × 10^3^	1.355 × 10^2^	7.853 × 10^1^	5.819 × 10^1^	4.122 × 10^3^	1.665 × 10^1^
F5	Ave	7.579 × 10^2^	1.138 × 10^3^	9.045 × 10^2^	8.460 × 10^2^	9.931 × 10^2^	7.246 × 10^2^	7.542 × 10^2^	9.341 × 10^2^	1.294 × 10^3^	6.295 × 10^2^
	Std	2.943 × 10^1^	1.143 × 10^2^	1.665 × 10^1^	3.125 × 10^1^	8.855 × 10^1^	3.277 × 10^1^	5.719 × 10^1^	2.413 × 10^1^	4.202 × 10^1^	1.776 × 10^1^
F6	Ave	6.252 × 10^2^	6.988 × 10^2^	6.652 × 10^2^	6.571 × 10^2^	6.662 × 10^2^	6.303 × 10^2^	6.323 × 10^2^	6.116 × 10^2^	6.960 × 10^2^	6.000 × 10^2^
	Std	4.540 × 10^0^	9.770 × 10^0^	6.534 × 10^0^	7.403 × 10^0^	8.921 × 10^0^	5.810 × 10^0^	8.340 × 10^0^	3.536 × 10^0^	4.591 × 10^0^	1.626 × 10^−12^
F7	Ave	1.179 × 10^3^	1.879 × 10^3^	1.795 × 10^3^	1.514 × 10^3^	1.375 × 10^3^	1.197 × 10^3^	1.109 × 10^3^	1.256 × 10^3^	4.877 × 10^3^	8.757 × 10^2^
	Std	9.683 × 10^1^	1.325 × 10^2^	5.528 × 10^1^	1.001 × 10^2^	1.383 × 10^2^	6.981 × 10^1^	6.477 × 10^1^	4.560 × 10^1^	3.155 × 10^2^	1.462 × 10^1^
F8	Ave	1.267 × 10^10^	2.122 × 10^10^	1.163 × 10^10^	2.011 × 10^8^	3.979 × 10^9^	6.726 × 10^8^	3.966 × 10^7^	1.968 × 10^8^	1.165 × 10^11^	8.776 × 10^2^
	Std	4.788 × 10^9^	5.243 × 10^9^	5.377 × 10^9^	2.236 × 10^8^	2.393 × 10^9^	3.594 × 10^8^	1.197 × 10^7^	9.826 × 10^7^	1.214 × 10^10^	8.751 × 10^2^
F9	Ave	1.239 × 10^4^	4.094 × 10^4^	2.720 × 10^4^	1.153 × 10^4^	2.882 × 10^4^	7.016 × 10^3^	1.187 × 10^4^	8.586 × 10^3^	4.859 × 10^4^	1.026 × 10^3^
	Std	5.348 × 10^3^	1.156 × 10^4^	6.628 × 10^3^	1.878 × 10^3^	7.571 × 10^3^	2.639 × 10^3^	5.839 × 10^3^	2.503 × 10^3^	5.081 × 10^3^	5.813 × 10^1^
F10	Ave	8.771 × 10^3^	1.313 × 10^4^	1.361 × 10^4^	9.013 × 10^3^	1.182 × 10^4^	9.832 × 10^3^	7.852 × 10^3^	1.362 × 10^4^	1.257 × 10^4^	4.834 × 10^3^
	Std	2.588 × 10^3^	8.816 × 10^2^	8.076 × 10^2^	1.829 × 10^3^	2.156 × 10^3^	2.885 × 10^3^	9.722 × 10^2^	5.457 × 10^2^	3.784 × 10^2^	3.539 × 10^2^
F11	Ave	7.984 × 10^3^	8.047 × 10^3^	5.854 × 10^3^	1.476 × 10^3^	5.096 × 10^3^	3.353 × 10^3^	1.785 × 10^3^	1.848 × 10^3^	3.687 × 10^4^	1.246 × 10^3^
	Std	3.353 × 10^3^	2.193 × 10^3^	2.997 × 10^3^	9.782 × 10^1^	2.682 × 10^3^	1.513 × 10^3^	1.309 × 10^2^	2.868 × 10^2^	1.053 × 10^4^	3.722 × 10^1^
F12	Ave	1.410 × 10^9^	4.823 × 10^9^	2.947 × 10^8^	2.049 × 10^7^	9.344 × 10^8^	8.076 × 10^7^	1.361 × 10^8^	2.277 × 10^7^	4.678 × 10^10^	2.405 × 10^6^
	Std	1.624 × 10^9^	2.316 × 10^9^	3.955 × 10^8^	1.264 × 10^7^	6.338 × 10^8^	9.878 × 10^7^	7.920 × 10^7^	1.107 × 10^7^	6.676 × 10^9^	1.146 × 10^6^
F13	Ave	2.724 × 10^8^	4.633 × 10^8^	2.362 × 10^6^	3.592 × 10^4^	1.066 × 10^8^	2.961 × 10^5^	6.657 × 10^5^	3.809 × 10^4^	3.517 × 10^10^	3.090 × 10^3^
	Std	2.656 × 10^8^	3.748 × 10^8^	3.063 × 10^6^	3.363 × 10^4^	1.225 × 10^8^	2.307 × 10^5^	9.836 × 10^5^	8.087 × 10^4^	6.786 × 10^9^	1.385 × 10^3^
F14	Ave	2.381 × 10^6^	8.468 × 10^6^	1.576 × 10^6^	1.169 × 10^5^	2.640 × 10^6^	6.983 × 10^5^	7.096 × 10^5^	1.207 × 10^5^	6.756 × 10^7^	1.558 × 10^5^
	Std	3.228 × 10^6^	6.772 × 10^6^	1.819 × 10^6^	9.670 × 10^4^	2.458 × 10^6^	7.179 × 10^5^	5.802 × 10^5^	9.758 × 10^4^	3.159 × 10^7^	1.365 × 10^5^
F15	Ave	1.836 × 10^7^	6.138 × 10^7^	8.305 × 10^4^	2.069 × 10^4^	2.400 × 10^7^	4.448 × 10^4^	1.204 × 10^5^	1.221 × 10^4^	1.487 × 10^10^	2.247 × 10^3^
	Std	3.234 × 10^7^	6.001 × 10^7^	3.817 × 10^4^	1.808 × 10^4^	8.113 × 10^7^	2.676 × 10^4^	5.769 × 10^4^	4.932 × 10^3^	5.438 × 10^9^	8.156 × 10^2^
F16	Ave	3.495 × 10^3^	6.466 × 10^3^	4.190 × 10^3^	3.577 × 10^3^	4.744 × 10^3^	3.445 × 10^3^	3.847 × 10^3^	4.544 × 10^3^	7.168 × 10^3^	2.693 × 10^3^
	Std	4.702 × 10^2^	1.194 × 10^3^	4.756 × 10^2^	6.395 × 10^2^	5.915 × 10^2^	3.448 × 10^2^	4.508 × 10^2^	3.445 × 10^2^	6.027 × 10^2^	2.729 × 10^2^
F17	Ave	3.024 × 10^3^	4.748 × 10^3^	3.619 × 10^3^	3.424 × 10^3^	4.235 × 10^3^	3.230 × 10^3^	3.376 × 10^3^	3.505 × 10^3^	6.115 × 10^5^	2.440 × 10^3^
	Std	3.831 × 10^2^	7.076 × 10^2^	3.599 × 10^2^	4.475 × 10^2^	4.606 × 10^2^	2.678 × 10^2^	3.479 × 10^2^	2.182 × 10^2^	4.480 × 10^5^	1.755 × 10^2^
F18	Ave	7.487 × 10^6^	6.934 × 10^7^	1.052 × 10^7^	7.387 × 10^5^	1.375 × 10^7^	3.802 × 10^6^	5.690 × 10^6^	2.560 × 10^6^	1.216 × 10^8^	4.299 × 10^5^
	Std	7.489 × 10^6^	5.714 × 10^7^	7.674 × 10^6^	5.095 × 10^5^	1.302 × 10^7^	2.832 × 10^6^	3.493 × 10^6^	2.369 × 10^6^	5.525 × 10^7^	2.296 × 10^5^
F19	Ave	6.869 × 10^6^	1.703 × 10^7^	3.451 × 10^5^	2.009 × 10^4^	8.627 × 10^6^	7.225 × 10^4^	6.701 × 10^5^	1.916 × 10^4^	7.629 × 10^9^	2.444 × 10^3^
	Std	1.014 × 10^7^	1.320 × 10^7^	3.290 × 10^5^	1.139 × 10^4^	1.012 × 10^7^	8.049 × 10^4^	5.013 × 10^5^	6.640 × 10^3^	1.817 × 10^9^	8.209 × 10^2^
F20	Ave	3.233 × 10^3^	3.988 × 10^3^	3.731 × 10^3^	3.264 × 10^3^	3.832 × 10^3^	3.257 × 10^3^	3.271 × 10^3^	3.703 × 10^3^	4.544 × 10^3^	2.562 × 10^3^
	Std	3.716 × 10^2^	3.324 × 10^2^	1.932 × 10^2^	2.776 × 10^2^	4.618 × 10^2^	4.577 × 10^2^	4.109 × 10^2^	1.984 × 10^2^	1.978 × 10^2^	1.744 × 10^2^
F21	Ave	2.561 × 10^3^	3.084 × 10^3^	2.739 × 10^3^	2.639 × 10^3^	2.882 × 10^3^	2.533 × 10^3^	2.556 × 10^3^	2.704 × 10^3^	3.088 × 10^3^	2.445 × 10^3^
	Std	5.955 × 10^1^	1.105 × 10^2^	9.385 × 10^1^	7.466 × 10^1^	9.423 × 10^1^	3.790 × 10^1^	5.475 × 10^1^	2.177 × 10^1^	3.794 × 10^1^	1.748 × 10^1^
F22	Ave	1.045 × 10^4^	1.475 × 10^4^	1.414 × 10^4^	1.134 × 10^4^	1.279 × 10^4^	1.127 × 10^4^	9.622 × 10^3^	1.287 × 10^4^	1.458 × 10^4^	6.723 × 10^3^
	Std	1.804 × 10^3^	8.218 × 10^2^	3.225 × 10^3^	1.686 × 10^3^	2.497 × 10^3^	2.531 × 10^3^	9.841 × 10^2^	4.985 × 10^3^	4.100 × 10^2^	4.031 × 10^2^
F23	Ave	3.073 × 10^3^	3.843 × 10^3^	3.284 × 10^3^	3.236 × 10^3^	3.571 × 10^3^	3.097 × 10^3^	3.050 × 10^3^	3.175 × 10^3^	3.509 × 10^3^	2.879 × 10^3^
	Std	8.909 × 10^1^	1.609 × 10^2^	1.143 × 10^2^	1.017 × 10^2^	1.377 × 10^2^	6.163 × 10^1^	6.015 × 10^1^	2.066 × 10^1^	3.632 × 10^1^	2.364 × 10^1^
F24	Ave	3.196 × 10^3^	3.947 × 10^3^	3.437 × 10^3^	3.353 × 10^3^	3.688 × 10^3^	3.228 × 10^3^	3.218 × 10^3^	3.341 × 10^3^	3.691 × 10^3^	3.158 × 10^3^
	Std	7.719 × 10^1^	1.590 × 10^2^	1.515 × 10^2^	1.191 × 10^2^	1.560 × 10^2^	7.256 × 10^1^	5.935 × 10^1^	3.550 × 10^1^	4.443 × 10^1^	6.566 × 10^1^
F25	Ave	3.923 × 10^3^	5.329 × 10^3^	3.941 × 10^3^	3.216 × 10^3^	3.848 × 10^3^	3.335 × 10^3^	3.154 × 10^3^	3.236 × 10^3^	2.442 × 10^4^	2.969 × 10^3^
	Std	4.657 × 10^2^	5.819 × 10^2^	3.694 × 10^2^	5.859 × 10^1^	1.401 × 10^3^	9.841 × 10^1^	5.135 × 10^1^	4.792 × 10^1^	4.560 × 10^3^	1.667 × 10^1^
F26	Ave	7.250 × 10^3^	1.534 × 10^4^	1.198 × 10^4^	9.027 × 10^3^	1.110 × 10^4^	7.808 × 10^3^	7.478 × 10^3^	7.988 × 10^3^	1.236 × 10^4^	5.317 × 10^3^
	Std	8.199 × 10^2^	1.550 × 10^3^	1.552 × 10^3^	2.245 × 10^3^	1.193 × 10^3^	7.051 × 10^2^	8.125 × 10^2^	1.805 × 10^3^	4.441 × 10^2^	1.915 × 10^2^
F27	Ave	3.729 × 10^3^	4.585 × 10^3^	3.872 × 10^3^	3.974 × 10^3^	4.096 × 10^3^	3.807 × 10^3^	3.614 × 10^3^	3.713 × 10^3^	3.941 × 10^3^	3.249 × 10^3^
	Std	1.182 × 10^2^	3.856 × 10^2^	1.927 × 10^2^	4.304 × 10^2^	3.297 × 10^2^	1.131 × 10^2^	9.897 × 10^1^	6.874 × 10^1^	9.115 × 10^1^	2.240 × 10^1^
F28	Ave	4.684 × 10^3^	6.019 × 10^3^	4.618 × 10^3^	3.645 × 10^3^	5.860 × 10^3^	4.079 × 10^3^	3.449 × 10^3^	3.710 × 10^3^	9.964 × 10^3^	3.263 × 10^3^
	Std	5.608 × 10^2^	5.809 × 10^2^	4.043 × 10^2^	1.155 × 10^2^	2.157 × 10^3^	2.657 × 10^2^	5.544 × 10^1^	1.184 × 10^2^	4.282 × 10^2^	5.789 × 10^0^
F29	Ave	4.955 × 10^3^	9.143 × 10^3^	5.468 × 10^3^	5.690 × 10^3^	6.070 × 10^3^	5.105 × 10^3^	5.159 × 10^3^	5.175 × 10^3^	2.185 × 10^4^	3.722 × 10^3^
	Std	5.070 × 10^2^	1.453 × 10^3^	5.696 × 10^2^	1.493 × 10^3^	9.336 × 10^2^	4.084 × 10^2^	4.263 × 10^2^	1.856 × 10^2^	1.841 × 10^4^	1.716 × 10^2^
F30	Ave	5.216 × 10^10^	1.095 × 10^11^	4.159 × 10^10^	1.589 × 10^10^	7.226 × 10^10^	1.541 × 10^10^	9.612 × 10^8^	1.309 × 10^10^	2.654 × 10^11^	9.484 × 10^2^
	Std	9.710 × 10^9^	1.254 × 10^10^	1.342 × 10^10^	6.663 × 10^9^	6.684 × 10^10^	3.519 × 10^9^	1.603 × 10^8^	4.352 × 10^9^	1.871 × 10^10^	9.550 × 10^2^

**Table 7:** Experimental results of CEC2017 (dim = 100).

Function	Metric	GWO	WOA	COA	IAGWO	DBO	SO	RIME	CPO	SPBO	ESPBO
F1	Ave	1.624 × 10^8^	3.430 × 10^8^	3.256 × 10^7^	2.317 × 10^6^	6.049 × 10^7^	1.469 × 10^7^	6.225 × 10^7^	9.859 × 10^6^	6.537 × 10^9^	6.673 × 10^5^
	Std	1.082 × 10^8^	1.487 × 10^8^	1.993 × 10^7^	1.210 × 10^6^	5.482 × 10^7^	8.589 × 10^6^	2.924 × 10^7^	4.047 × 10^6^	9.302 × 10^8^	6.531 × 10^4^
F2	Ave	1.491 × 10^136^	3.509 × 10^177^	5.566 × 10^151^	2.746 × 10^143^	9.998 × 10^155^	2.764 × 10^138^	5.330 × 10^123^	2.720 × 10^121^	1.129 × 10^156^	4.566 × 10^39^
	Std	8.168 × 10^136^	6.554 × 10^4^	3.049 × 10^152^	1.504 × 10^144^	6.554 × 10^4^	1.483 × 10^139^	2.919 × 10^124^	1.153 × 10^122^	6.554 × 10^4^	1.663 × 10^40^
F3	Ave	5.480 × 10^5^	9.416 × 10^5^	7.075 × 10^5^	2.496 × 10^5^	6.466 × 10^5^	3.687 × 10^5^	7.035 × 10^5^	4.655 × 10^5^	7.781 × 10^5^	7.310 × 10^5^
	Std	8.773 × 10^4^	1.810 × 10^5^	1.300 × 10^5^	2.435 × 10^4^	3.108 × 10^5^	3.167 × 10^4^	1.180 × 10^5^	4.906 × 10^4^	6.528 × 10^4^	7.069 × 10^4^
F4	Ave	5.614 × 10^3^	2.233 × 10^4^	8.720 × 10^3^	2.668 × 10^3^	1.488 × 10^4^	2.620 × 10^3^	1.221 × 10^3^	2.516 × 10^3^	5.714 × 10^4^	5.900 × 10^2^
	Std	1.477 × 10^3^	4.884 × 10^3^	2.660 × 10^3^	9.966 × 10^2^	1.395 × 10^4^	5.238 × 10^2^	1.344 × 10^2^	4.330 × 10^2^	6.673 × 10^3^	2.101 × 10^1^
F5	Ave	1.232 × 10^3^	1.959 × 10^3^	1.538 × 10^3^	1.415 × 10^3^	1.689 × 10^3^	1.175 × 10^3^	1.318 × 10^3^	1.671 × 10^3^	2.191 × 10^3^	9.386 × 10^2^
	Std	6.368 × 10^1^	1.301 × 10^2^	5.246 × 10^1^	5.049 × 10^1^	2.329 × 10^2^	6.027 × 10^1^	1.015 × 10^2^	5.245 × 10^1^	6.170 × 10^1^	3.816 × 10^1^
F6	Ave	6.460 × 10^2^	7.075 × 10^2^	6.733 × 10^2^	6.686 × 10^2^	6.781 × 10^2^	6.474 × 10^2^	6.563 × 10^2^	6.427 × 10^2^	7.020 × 10^2^	6.000 × 10^2^
	Std	4.977 × 10^0^	9.871 × 10^0^	3.499 × 10^0^	4.011 × 10^0^	1.060 × 10^1^	7.709 × 10^0^	6.075 × 10^0^	5.408 × 10^0^	3.573 × 10^0^	1.900 × 10^−13^
F7	Ave	2.233 × 10^3^	3.828 × 10^3^	3.370 × 10^3^	3.110 × 10^3^	2.938 × 10^3^	2.266 × 10^3^	2.197 × 10^3^	2.360 × 10^3^	9.563 × 10^3^	1.258 × 10^3^
	Std	1.319 × 10^2^	1.302 × 10^2^	1.159 × 10^2^	1.353 × 10^2^	1.815 × 10^2^	1.318 × 10^2^	1.997 × 10^2^	1.045 × 10^2^	7.163 × 10^2^	2.551 × 10^1^
F8	Ave	1.569 × 10^3^	2.403 × 10^3^	2.040 × 10^3^	1.815 × 10^3^	2.162 × 10^3^	1.502 × 10^3^	1.627 × 10^3^	1.969 × 10^3^	2.476 × 10^3^	1.244 × 10^3^
	Std	9.938 × 10^1^	1.512 × 10^2^	4.888 × 10^1^	7.773 × 10^1^	2.270 × 10^2^	7.106 × 10^1^	1.143 × 10^2^	4.705 × 10^1^	7.114 × 10^1^	3.624 × 10^1^
F9	Ave	4.336 × 10^4^	8.060 × 10^4^	5.028 × 10^4^	2.854 × 10^4^	7.373 × 10^4^	2.980 × 10^4^	5.269 × 10^4^	4.863 × 10^4^	1.101 × 10^5^	8.077 × 10^3^
	Std	1.403 × 10^4^	1.711 × 10^4^	1.392 × 10^4^	2.207 × 10^3^	1.448 × 10^4^	6.693 × 10^3^	1.907 × 10^4^	6.754 × 10^3^	7.446 × 10^3^	6.185 × 10^3^
F10	Ave	2.126 × 10^4^	2.953 × 10^4^	2.415 × 10^4^	1.896 × 10^4^	2.786 × 10^4^	3.164 × 10^4^	1.933 × 10^4^	3.080 × 10^4^	2.646 × 10^4^	1.130 × 10^4^
	Std	5.116 × 10^10^	1.095 × 10^11^	7.179 × 10^10^	1.589 × 10^10^	7.226 × 10^10^	1.541 × 10^10^	9.612 × 10^8^	1.309 × 10^10^	2.654 × 10^11^	9.484 × 10^2^
F11	Ave	8.706 × 10^4^	3.152 × 10^5^	3.109 × 10^5^	3.743 × 10^4^	2.275 × 10^5^	1.342 × 10^5^	4.004 × 10^4^	9.296 × 10^4^	2.121 × 10^5^	3.187 × 10^4^
	Std	1.567 × 10^4^	1.300 × 10^5^	9.415 × 10^4^	9.843 × 10^3^	6.421 × 10^4^	2.515 × 10^4^	1.158 × 10^4^	1.647 × 10^4^	3.825 × 10^4^	1.934 × 10^4^
F12	Ave	1.167 × 10^10^	3.045 × 10^10^	1.056 × 10^10^	6.776 × 10^8^	7.520 × 10^9^	1.731 × 10^9^	1.079 × 10^9^	9.167 × 10^8^	1.416 × 10^11^	1.140 × 10^7^
	Std	6.818 × 10^9^	6.479 × 10^9^	4.912 × 10^9^	2.307 × 10^8^	2.976 × 10^9^	8.344 × 10^8^	4.523 × 10^8^	2.776 × 10^8^	1.708 × 10^10^	3.693 × 10^6^
F13	Ave	1.443 × 10^9^	3.102 × 10^9^	7.210 × 10^8^	1.699 × 10^5^	3.205 × 10^8^	6.072 × 10^6^	1.995 × 10^6^	2.236 × 10^5^	4.017 × 10^10^	3.258 × 10^3^
	Std	1.121 × 10^9^	1.334 × 10^9^	8.901 × 10^8^	1.749 × 10^5^	2.356 × 10^8^	9.401 × 10^6^	6.679 × 10^5^	1.635 × 10^5^	4.519 × 10^9^	1.239 × 10^3^
F14	Ave	8.182 × 10^6^	2.158 × 10^7^	1.339 × 10^7^	1.212 × 10^6^	1.578 × 10^7^	7.836 × 10^6^	8.408 × 10^6^	4.340 × 10^6^	1.857 × 10^8^	1.753 × 10^6^
	Std	3.203 × 10^6^	1.241 × 10^7^	8.548 × 10^6^	5.293 × 10^5^	1.123 × 10^7^	3.753 × 10^6^	3.116 × 10^6^	1.984 × 10^6^	6.262 × 10^7^	9.600 × 10^5^
F15	Ave	3.331 × 10^8^	5.625 × 10^8^	1.316 × 10^7^	3.130 × 10^4^	1.241 × 10^8^	3.347 × 10^5^	1.574 × 10^6^	1.023 × 10^4^	1.914 × 10^10^	2.288 × 10^3^
	Std	4.215 × 10^8^	4.344 × 10^8^	4.045 × 10^7^	1.570 × 10^4^	1.801 × 10^8^	2.668 × 10^5^	4.356 × 10^6^	3.097 × 10^3^	3.102 × 10^9^	3.067 × 10^2^
F16	Ave	6.864 × 10^3^	1.642 × 10^4^	8.674 × 10^3^	6.879 × 10^3^	9.351 × 10^3^	7.240 × 10^3^	7.386 × 10^3^	1.013 × 10^4^	1.640 × 10^4^	4.577 × 10^3^
	Std	8.988 × 10^2^	2.087 × 10^3^	1.307 × 10^3^	8.106 × 10^2^	1.362 × 10^3^	1.366 × 10^3^	8.555 × 10^2^	5.074 × 10^2^	1.299 × 10^3^	4.121 × 10^2^
F17	Ave	5.687 × 10^3^	3.095 × 10^4^	7.004 × 10^3^	6.028 × 10^3^	8.811 × 10^3^	5.762 × 10^3^	5.717 × 10^3^	7.050 × 10^3^	2.772 × 10^6^	4.009 × 10^3^
	Std	1.360 × 10^3^	4.426 × 10^4^	1.241 × 10^3^	6.883 × 10^2^	1.078 × 10^3^	5.581 × 10^2^	6.757 × 10^2^	3.144 × 10^2^	1.365 × 10^6^	3.597 × 10^2^
F18	Ave	1.031 × 10^7^	2.090 × 10^7^	1.006 × 10^7^	2.058 × 10^6^	2.228 × 10^7^	9.054 × 10^6^	1.048 × 10^7^	4.468 × 10^6^	1.716 × 10^8^	1.728 × 10^6^
	Std	7.727 × 10^6^	1.141 × 10^7^	8.588 × 10^6^	1.007 × 10^6^	1.699 × 10^7^	3.714 × 10^6^	5.166 × 10^6^	2.240 × 10^6^	5.045 × 10^7^	7.168 × 10^5^
F19	Ave	3.236 × 10^8^	4.985 × 10^8^	2.061 × 10^7^	8.969 × 10^4^	8.349 × 10^7^	2.220 × 10^6^	2.052 × 10^7^	1.199 × 10^4^	2.118 × 10^10^	2.784 × 10^3^
	Std	4.746 × 10^8^	3.054 × 10^8^	1.683 × 10^7^	8.931 × 10^4^	6.739 × 10^7^	1.284 × 10^6^	1.267 × 10^7^	4.909 × 10^3^	3.043 × 10^9^	7.892 × 10^2^
F20	Ave	5.456 × 10^3^	7.257 × 10^3^	6.885 × 10^3^	5.527 × 10^3^	7.209 × 10^3^	7.140 × 10^3^	5.814 × 10^3^	7.248 × 10^3^	7.862 × 10^3^	4.258 × 10^3^
	Std	9.634 × 10^2^	5.371 × 10^2^	6.070 × 10^2^	5.245 × 10^2^	8.133 × 10^2^	4.059 × 10^2^	5.939 × 10^2^	4.008 × 10^2^	4.045 × 10^2^	3.678 × 10^2^
F21	Ave	3.115 × 10^3^	4.442 × 10^3^	3.753 × 10^3^	3.504 × 10^3^	4.005 × 10^3^	3.099 × 10^3^	3.160 × 10^3^	3.400 × 10^3^	4.146 × 10^3^	2.776 × 10^3^
	Std	1.524 × 10^2^	1.870 × 10^2^	1.545 × 10^2^	1.471 × 10^2^	1.797 × 10^2^	8.614 × 10^1^	8.144 × 10^1^	3.577 × 10^1^	6.999 × 10^1^	3.929 × 10^1^
F22	Ave	2.426 × 10^4^	3.201 × 10^4^	2.954 × 10^4^	2.422 × 10^4^	3.061 × 10^4^	3.275 × 10^4^	2.283 × 10^4^	3.314 × 10^4^	2.839 × 10^4^	1.357 × 10^4^
	Std	5.884 × 10^3^	1.648 × 10^3^	2.645 × 10^3^	3.617 × 10^3^	4.451 × 10^3^	2.062 × 10^3^	1.476 × 10^3^	6.464 × 10^2^	3.825 × 10^2^	7.283 × 10^2^
F23	Ave	3.714 × 10^3^	5.315 × 10^3^	4.304 × 10^3^	4.190 × 10^3^	4.805 × 10^3^	3.667 × 10^3^	3.684 × 10^3^	4.004 × 10^3^	4.278 × 10^3^	3.079 × 10^3^
	Std	1.158 × 10^2^	2.680 × 10^2^	1.804 × 10^2^	1.884 × 10^2^	2.207 × 10^2^	9.883 × 10^1^	1.230 × 10^2^	4.653 × 10^1^	4.358 × 10^1^	2.891 × 10^1^
F24	Ave	4.528 × 10^3^	6.738 × 10^3^	5.377 × 10^3^	5.087 × 10^3^	5.996 × 10^3^	4.768 × 10^3^	4.182 × 10^3^	4.554 × 10^3^	4.990 × 10^3^	3.710 × 10^3^
	Std	1.474 × 10^2^	4.157 × 10^2^	2.680 × 10^2^	4.251 × 10^2^	3.965 × 10^2^	2.566 × 10^2^	1.241 × 10^2^	8.167 × 10^1^	7.158 × 10^1^	3.517 × 10^1^
F25	Ave	7.205 × 10^3^	1.096 × 10^4^	8.209 × 10^3^	4.918 × 10^3^	9.606 × 10^3^	5.580 × 10^3^	3.970 × 10^3^	4.976 × 10^3^	5.912 × 10^4^	3.165 × 10^3^
	Std	1.016 × 10^3^	9.705 × 10^2^	9.918 × 10^2^	3.471 × 10^2^	6.347 × 10^3^	5.112 × 10^2^	1.347 × 10^2^	3.102 × 10^2^	6.062 × 10^3^	4.972 × 10^1^
F26	Ave	1.771 × 10^4^	3.911 × 10^4^	3.195 × 10^4^	2.648 × 10^4^	2.669 × 10^4^	2.013 × 10^4^	1.601 × 10^4^	2.113 × 10^4^	2.317 × 10^4^	1.054 × 10^4^
	Std	2.199 × 10^3^	2.825 × 10^3^	3.400 × 10^3^	3.824 × 10^3^	3.923 × 10^3^	1.633 × 10^3^	1.580 × 10^3^	1.502 × 10^3^	5.254 × 10^2^	4.546 × 10^2^
F27	Ave	4.309 × 10^3^	6.498 × 10^3^	4.439 × 10^3^	4.205 × 10^3^	4.806 × 10^3^	4.363 × 10^3^	4.055 × 10^3^	4.256 × 10^3^	4.367 × 10^3^	3.360 × 10^3^
	Std	1.805 × 10^2^	1.124 × 10^3^	3.314 × 10^2^	3.650 × 10^2^	4.448 × 10^2^	1.508 × 10^2^	2.040 × 10^2^	1.257 × 10^2^	1.314 × 10^2^	3.428 × 10^1^
F28	Ave	9.662 × 10^3^	1.470 × 10^4^	1.146 × 10^4^	5.266 × 10^3^	1.745 × 10^4^	9.768 × 10^3^	4.318 × 10^3^	6.069 × 10^3^	2.162 × 10^4^	3.342 × 10^3^
	Std	1.470 × 10^3^	1.126 × 10^3^	1.520 × 10^3^	6.313 × 10^2^	6.710 × 10^3^	1.496 × 10^3^	3.270 × 10^2^	6.132 × 10^2^	1.372 × 10^3^	9.850 × 10^0^
F29	Ave	9.254 × 10^3^	2.014 × 10^4^	1.182 × 10^4^	8.644 × 10^3^	1.182 × 10^4^	9.124 × 10^3^	9.596 × 10^3^	1.027 × 10^4^	7.107 × 10^5^	5.936 × 10^3^
	Std	8.424 × 10^2^	4.744 × 10^3^	1.295 × 10^3^	8.525 × 10^2^	2.200 × 10^3^	9.636 × 10^2^	7.880 × 10^2^	4.768 × 10^2^	4.075 × 10^5^	4.457 × 10^2^
F30	Ave	1.480 × 10^9^	3.300 × 10^9^	6.562 × 10^8^	6.031 × 10^6^	2.488 × 10^8^	2.277 × 10^7^	1.744 × 10^8^	7.063 × 10^6^	1.575 × 10^10^	8.574 × 10^3^
	Std	1.215 × 10^9^	1.437 × 10^9^	1.309 × 10^9^	3.197 × 10^6^	1.312 × 10^8^	1.763 × 10^7^	7.082 × 10^7^	4.094 × 10^6^	2.240 × 10^9^	1.781 × 10^3^

**Table 8:** Experimental results of CEC2020 (dim = 20).

Function	Metric	GWO	WOA	COA	IAGWO	DBO	SO	RIME	CPO	SPBO	ESPBO
F1	Ave	1.026 × 10^9^	1.213 × 10^9^	7.335 × 10^7^	2.655 × 10^3^	3.185 × 10^7^	7.230 × 10^4^	5.224 × 10^5^	8.467 × 10^3^	6.502 × 10^2^	3.474 × 10^2^
	Std	1.107 × 10^9^	9.991 × 10^8^	2.100 × 10^8^	3.034 × 10^3^	3.047 × 10^7^	6.206 × 10^4^	3.237 × 10^5^	6.189 × 10^3^	2.805 × 10^2^	3.336 × 10^1^
F2	Ave	2.648 × 10^3^	4.278 × 10^3^	4.249 × 10^3^	3.415 × 10^3^	3.451 × 10^3^	2.122 × 10^3^	1.997 × 10^3^	3.271 × 10^3^	5.266 × 10^3^	1.252 × 10^3^
	Std	4.362 × 10^2^	7.370 × 10^2^	7.299 × 10^2^	5.809 × 10^2^	6.640 × 10^2^	3.200 × 10^2^	3.413 × 10^2^	2.317 × 10^2^	3.253 × 10^2^	8.249 × 10^1^
F3	Ave	7.857 × 10^2^	9.667 × 10^2^	9.194 × 10^2^	8.552 × 10^2^	8.387 × 10^2^	8.074 × 10^2^	7.712 × 10^2^	7.850 × 10^2^	2.050 × 10^3^	7.218 × 10^2^
	Std	2.434 × 10^1^	4.986 × 10^1^	5.056 × 10^1^	3.482 × 10^1^	4.137 × 10^1^	3.306 × 10^1^	1.522 × 10^1^	7.205 × 10^0^	1.332 × 10^2^	5.886 × 10^0^
F4	Ave	1.937 × 10^3^	3.044 × 10^3^	1.923 × 10^3^	1.925 × 10^3^	1.925 × 10^3^	1.908 × 10^3^	1.906 × 10^3^	1.908 × 10^3^	2.258 × 10^5^	1.901 × 10^3^
	Std	8.066 × 10^1^	2.885 × 10^3^	8.557 × 10^0^	1.776 × 10^1^	1.336 × 10^1^	2.765 × 10^0^	1.460 × 10^0^	9.893 × 10^−1^	1.363 × 10^5^	1.762 × 10^−1^
F5	Ave	1.247 × 10^6^	2.894 × 10^6^	6.344 × 10^5^	4.812 × 10^4^	9.505 × 10^5^	2.811 × 10^5^	3.171 × 10^5^	2.921 × 10^4^	3.224 × 10^7^	7.813 × 10^4^
	Std	1.130 × 10^6^	2.168 × 10^6^	3.774 × 10^5^	3.561 × 10^4^	7.879 × 10^5^	1.995 × 10^5^	2.456 × 10^5^	2.184 × 10^4^	1.555 × 10^7^	9.253 × 10^4^
F6	Ave	1.232 × 10^3^	1.929 × 10^3^	1.538 × 10^3^	1.415 × 10^3^	1.637 × 10^3^	1.134 × 10^3^	1.372 × 10^3^	1.641 × 10^3^	2.199 × 10^3^	9.416 × 10^2^
	Std	6.164 × 10^2^	2.695 × 10^2^	4.506 × 10^2^	7.334 × 10^0^	6.043 × 10^2^	1.207 × 10^0^	8.677 × 10^2^	1.363 × 10^1^	9.281 × 10^2^	2.216 × 10^1^
F7	Ave	5.620 × 10^5^	1.793 × 10^6^	4.954 × 10^5^	1.419 × 10^4^	2.839 × 10^5^	1.305 × 10^5^	2.142 × 10^5^	4.420 × 10^3^	1.802 × 10^7^	4.811 × 10^4^
	Std	1.143 × 10^6^	1.653 × 10^6^	5.101 × 10^5^	1.434 × 10^4^	2.147 × 10^5^	1.459 × 10^5^	2.223 × 10^5^	1.125 × 10^3^	1.271 × 10^7^	9.351 × 10^4^
F8	Ave	3.060 × 10^3^	4.522 × 10^3^	2.324 × 10^3^	2.302 × 10^3^	2.850 × 10^3^	2.656 × 10^3^	3.014 × 10^3^	2.300 × 10^3^	6.420 × 10^3^	2.809 × 10^3^
	Std	1.059 × 10^3^	1.876 × 10^3^	7.421 × 10^1^	3.672 × 10^0^	1.147 × 10^3^	7.398 × 10^2^	1.126 × 10^3^	2.691 × 10^−1^	5.235 × 10^2^	6.925 × 10^2^
F9	Ave	2.885 × 10^3^	3.029 × 10^3^	2.885 × 10^3^	2.932 × 10^3^	3.010 × 10^3^	2.866 × 10^3^	2.870 × 10^3^	2.889 × 10^3^	2.481 × 10^3^	2.481 × 10^3^
	Std	4.366 × 10^1^	7.502 × 10^1^	2.637 × 10^1^	6.149 × 10^1^	6.553 × 10^1^	2.414 × 10^1^	2.738 × 10^1^	1.655 × 10^1^	2.146 × 10^1^	2.988 × 10^−4^
F10	Ave	3.015 × 10^3^	3.132 × 10^3^	2.991 × 10^3^	2.982 × 10^3^	2.991 × 10^3^	2.962 × 10^3^	2.954 × 10^3^	2.964 × 10^3^	6.918 × 10^3^	2.908 × 10^3^
	Std	4.111 × 10^1^	7.939 × 10^1^	2.565 × 10^1^	3.402 × 10^1^	5.610 × 10^1^	3.356 × 10^1^	3.387 × 10^1^	3.099 × 10^1^	1.261 × 10^3^	4.327 × 10^0^

**Table 9:** Summary of Wilcoxon rank-sum test results (α = 0.05) across all 112 benchmark functions.

Benchmark Suite	Dimension	Functions	Bold (*p* ≥ 0.05)	+(Significantly Better, *p* < 0.05)	≈(No Significant Difference, *p* ≥ 0.05)	−(Significantly Worse)	Win Rate (%)
CEC2022	20	12	10	98	10	0	90.7
CEC2020	20	10	6	84	6	0	93.3
CEC2017	30	30	20	250	20	0	92.6
CEC2017	50	30	25	245	25	0	90.7
CEC2017	100	30	23	247	23	0	91.5
OVERALL	-	112	84	924	84	0	91.7

**Table 10:** Friedman mean rank test results.

Suites	CEC2017				CEC2022				CEC2020	
Dim	30		50		100		20		20	
Algorithms	M_R	T_R	M_R	T_R	M_R	T_R	M_R	T_R	M_R	T_R
GWO	5.35	6	5.06	6	4.77	5	5.62	6	5.50	6
WOA	8.87	9	8.90	9	8.90	10	9.08	10	8.30	9
COA	6.29	7	6.65	7	6.65	7	6.31	7	6.20	7
IAGWO	3.55	2	3.23	2	2.39	2	4.31	5	3.80	3
DBO	7.16	8	7.06	8	7.29	8	6.85	8	6.90	8
SO	3.97	5	3.87	3	4.55	4	4.08	4	4.10	4
RIME	3.94	4	3.87	4	4.10	3	3.54	3	4.40	5
CPO	3.65	3	4.32	5	4.81	6	8.08	9	3.80	2
SPBO	9.58	10	9.39	10	8.87	9	2.46	2	9.90	10
ESPBO	1.19	1	1.19	1	1.23	1	1.23	1	2.10	1

**Table 11:** Performance results of UAV 3D trajectory planning experiments.

Algorithms	Best	Ave	Worst	Std	Rank
GWO	209.4727	232.2354	404.4066	36.8602	5
WOA	278.8787	306.2781	736.6825	97.1586	10
COA	221.4765	284.4462	535.1926	59.7469	9
GTO	262.3810	250.6849	443.6914	65.6775	7
DBO	211.1476	249.2114	432.7954	50.0317	6
SO	224.5803	267.4475	462.8923	64.7611	8
RIME	214.5172	207.8842	220.9472	7.1473	2
CPO	223.2291	211.7805	302.7988	24.1273	3
GOA	205.6108	221.2846	256.3767	10.0902	4
ESPBO	199.8874	205.8179	227.2393	5.3440	1

## Data Availability

All data in this paper are included in the manuscript.

## Appendix A

**Table A1:** Statistical *p*-values for 9 algorithms in the CEC-2022 (D = 20) evaluation.

Function/ Algorithm	GWO	WOA	COA	IAGWO	DBO	RIME	SO	CPO	SPBO
F1	3.643 × 10^−8^	5.970 × 10^−9^	9.760 × 10^−10^	6.283 × 10^−6^	1.194 × 10^−6^	6.696 × 10^−11^	6.700 × 10^−11^	5.020 × 10^−11^	7.283 × 10^−1^
F2	3.338 × 10^−11^	3.340 × 10^−11^	3.725 × 10^−7^	3.474 × 10^−10^	2.371 × 10^−10^	4.077 × 10^−11^	4.931 × 10^−4^	4.440 × 10^−7^	3.020 × 10^−11^
F3	7.770 × 10^−9^	1.960 × 10^−10^	2.153 × 10^−10^	4.931 × 10^−4^	7.632 × 10^−11^	3.592 × 10^−5^	3.081 × 10^−8^	7.390 × 10^−11^	3.255 × 10^−1^
F4	3.520 × 10^−7^	2.390 × 10^−8^	3.820 × 10^−10^	4.113 × 10^−7^	3.690 × 10^−11^	8.485 × 10^−9^	4.063 × 10^−11^	2.372 × 10^−10^	4.113 × 10^−7^
F5	1.194 × 10^−6^	5.573 × 10^−10^	4.200 × 10^−10^	3.988 × 10^−4^	6.066 × 10^−11^	1.729 × 10^−7^	7.380 × 10^−10^	7.013 × 10^−2^	7.283 × 10^−1^
F6	6.528 × 10^−8^	3.555 × 10^−1^	8.315 × 10^−3^	4.504 × 10^−11^	3.579 × 10^−11^	7.659 × 10^−5^	3.338 × 10^−11^	3.952 × 10^−8^	1.302 × 10^−3^
F7	1.695 × 10^−9^	7.389 × 10^−11^	2.610 × 10^−10^	4.427 × 10^−3^	1.464 × 10^−10^	4.183 × 10^−9^	9.919 × 10^−11^	2.783 × 10^−7^	3.555 × 10^−1^
F8	4.200 × 10^−10^	4.077 × 10^−11^	3.183 × 10^−3^	5.072 × 10^−10^	3.835 × 10^−6^	1.957 × 10^−10^	5.494 × 10^−11^	1.070 × 10^−9^	3.690 × 10^−11^
F9	1.174 × 10^−9^	1.011 × 10^−8^	1.120 × 10^−1^	4.113 × 10^−7^	1.596 × 10^−7^	4.358 × 10^−2^	3.690 × 10^−11^	9.756 × 10^−10^	9.705 × 10^−1^
F10	1.254 × 10^−7^	4.504 × 10^−11^	5.600 × 10^−7^	3.871 × 10^−1^	4.444 × 10^−7^	2.879 × 10^−6^	2.872 × 10^−10^	5.265 × 10^−5^	1.023 × 10^−1^
F11	1.287 × 10^−9^	4.077 × 10^−11^	2.278 × 10^−5^	1.385 × 10^−6^	1.957 × 10^−10^	9.919 × 10^−11^	1.695 × 10^−9^	6.528 × 10^−8^	3.338 × 10^−11^
F12	3.470 × 10^−10^	3.070 × 10^−11^	9.760 × 10^−10^	1.780 × 10^−10^	1.910 × 10^−2^	1.090 × 10^−10^	2.370 × 10^−10^	3.690 × 10^−11^	2.380 × 10^−7^

**Table A2:** Statistical *p*-values for 9 algorithms in the CEC-2017 (D = 30) evaluation.

Function/ Algorithm	GWO	WOA	COA	IAGWO	DBO	RIME	SO	CPO	SPBO
F1	8.459 × 10^−9^	9.563 × 10^−5^	7.482 × 10^−7^	3.957 × 10^−6^	7.415 × 10^−3^	1.729 × 10^−7^	9.563 × 10^−5^	4.616 × 10^−10^	6.567 × 10^−2^
F2	9.147 × 10^−6^	6.789 × 10^−6^	3.456 × 10^−4^	9.234 × 10^−4^	7.891 × 10^−8^	6.722 × 10^−10^	6.789 × 10^−6^	3.035 × 10^−11^	4.616 × 10^−10^
F3	1.349 × 10^−9^	4.200 × 10^−10^	3.590 × 10^−5^	2.920 × 10^−9^	2.530 × 10^−11^	3.027 × 10^−11^	5.490 × 10^−11^	3.018 × 10^−11^	3.350 × 10^−8^
F4	4.500 × 10^−11^	3.976 × 10^−11^	1.600 × 10^−7^	1.160 × 10^−7^	1.910 × 10^−2^	4.690 × 10^−8^	3.830 × 10^−5^	3.340 × 10^−11^	6.360 × 10^−5^
F5	2.870 × 10^−10^	6.120 × 10^−10^	1.330 × 10^−10^	2.094 × 10^−8^	8.453 × 10^−11^	7.380 × 10^−10^	1.950 × 10^−3^	4.080 × 10^−11^	9.830 × 10^−8^
F6	8.880 × 10^−6^	3.107 × 10^−9^	1.472 × 10^−7^	3.081 × 10^−8^	1.038 × 10^−9^	1.596 × 10^−7^	3.643 × 10^−8^	1.410 × 10^−9^	8.236 × 10^−2^
F7	3.068 × 10^−11^	2.154 × 10^−10^	4.214 × 10^−1^	1.698 × 10^−8^	5.494 × 10^−11^	1.206 × 10^−10^	6.722 × 10^−10^	6.121 × 10^−10^	1.453 × 10^−1^
F8	9.756 × 10^−10^	6.121 × 10^−10^	4.975 × 10^−11^	2.195 × 10^−8^	3.720 × 10^−11^	1.385 × 10^−6^	4.226 × 10^−3^	1.957 × 10^−10^	2.670 × 10^−9^
F9	1.777 × 10^−10^	6.518 × 10^−9^	7.020 × 10^−11^	5.462 × 10^−9^	9.919 × 10^−11^	9.514 × 10^−6^	5.573 × 10^−10^	4.077 × 10^−11^	7.062 × 10^−1^
F10	1.911 × 10^−2^	9.260 × 10^−9^	3.690 × 10^−11^	8.841 × 10^−7^	4.975 × 10^−11^	1.174 × 10^−9^	4.311 × 10^−8^	1.606 × 10^−6^	1.777 × 10^−10^
F11	1.094 × 10^−10^	3.825 × 10^−9^	1.206 × 10^−10^	1.206 × 10^−10^	8.153 × 10^−11^	2.389 × 10^−4^	5.264 × 10^−4^	6.066 × 10^−11^	3.690 × 10^−11^
F12	2.002 × 10^−6^	8.485 × 10^−9^	2.254 × 10^−4^	5.627 × 10^−11^	4.084 × 10^−5^	7.389 × 10^−11^	1.606 × 10^−6^	4.975 × 10^−11^	8.993 × 10^−11^
F13	1.547 × 10^−9^	8.891 × 10^−10^	2.572 × 10^−7^	3.210 × 10^−11^	1.114 × 10^−3^	2.390 × 10^−8^	2.254 × 10^−4^	1.206 × 10^−10^	5.573 × 10^−10^
F14	3.068 × 10^−11^	2.154 × 10^−10^	4.204 × 10^−1^	1.698 × 10^−8^	5.494 × 10^−11^	1.206 × 10^−10^	6.722 × 10^−10^	6.121 × 10^−10^	1.453 × 10^−1^
F15	6.159 × 10^−2^	2.847 × 10^−7^	6.284 × 10^−10^	8.923 × 10^−2^	1.087 × 10^−10^	4.352 × 10^−11^	5.738 × 10^−4^	5.234 × 10^−7^	4.077 × 10^−11^
F16	8.765 × 10^−11^	3.456 × 10^−7^	1.234 × 10^−10^	4.257 × 10^−4^	7.389 × 10^−11^	1.606 × 10^−6^	4.182 × 10^−8^	9.260 × 10^−11^	7.389 × 10^−11^
F17	9.563 × 10^−7^	2.369 × 10^−2^	8.427 × 10^−10^	9.347 × 10^−5^	2.345 × 10^−9^	2.195 × 10^−7^	4.257 × 10^−8^	1.729 × 10^−9^	7.291 × 10^−1^
F18	3.289 × 10^−2^	4.562 × 10^−7^	9.234 × 10^−10^	5.426 × 10^−6^	5.967 × 10^−10^	2.416 × 10^−10^	4.352 × 10^−11^	8.533 × 10^−8^	3.820 × 10^−10^
F19	7.415 × 10^−8^	4.139 × 10^−7^	6.284 × 10^−10^	2.583 × 10^−4^	4.077 × 10^−11^	2.345 × 10^−6^	7.172 × 10^−5^	5.592 × 10^−10^	9.514 × 10^−6^
F20	5.189 × 10^−7^	6.129 × 10^−6^	3.957 × 10^−10^	6.285 × 10^−6^	9.234 × 10^−11^	3.456 × 10^−10^	4.077 × 10^−11^	5.967 × 10^−11^	4.139 × 10^−1^
F21	9.876 × 10^−8^	2.345 × 10^−7^	8.765 × 10^−10^	4.352 × 10^−4^	1.234 × 10^−10^	4.686 × 10^−9^	1.234 × 10^−7^	9.234 × 10^−11^	4.616 × 10^−10^
F22	7.291 × 10^−5^	5.189 × 10^−5^	3.718 × 10^−6^	9.049 × 10^−2^	1.234 × 10^−7^	7.695 × 10^−8^	1.373 × 10^−1^	5.234 × 10^−2^	8.534 × 10^−1^
F23	4.182 × 10^−8^	1.478 × 10^−6^	9.234 × 10^−4^	7.482 × 10^−7^	8.427 × 10^−3^	3.197 × 10^−9^	1.478 × 10^−6^	7.482 × 10^−10^	7.845 × 10^−1^
F24	2.485 × 10^−6^	5.731 × 10^−5^	7.415 × 10^−7^	3.456 × 10^−4^	7.815 × 10^−8^	3.690 × 10^−11^	5.731 × 10^−5^	4.686 × 10^−8^	4.077 × 10^−11^
F25	4.257 × 10^−9^	6.789 × 10^−6^	2.345 × 10^−4^	5.189 × 10^−6^	5.731 × 10^−3^	2.872 × 10^−10^	6.789 × 10^−6^	2.278 × 10^−5^	7.389 × 10^−11^
F26	3.749 × 10^−10^	8.346 × 10^−5^	4.290 × 10^−1^	2.198 × 10^−4^	5.432 × 10^−8^	3.324 × 10^−6^	8.346 × 10^−5^	4.200 × 10^−10^	5.997 × 10^−1^
F27	8.163 × 10^−8^	1.478 × 10^−6^	8.923 × 10^−4^	8.179 × 10^−7^	5.234 × 10^−3^	9.260 × 10^−9^	1.478 × 10^−6^	5.967 × 10^−9^	3.820 × 10^−10^
F28	5.738 × 10^−7^	5.731 × 10^−5^	4.352 × 10^−6^	6.789 × 10^−4^	4.562 × 10^−8^	2.597 × 10^−5^	5.731 × 10^−5^	8.766 × 10^−1^	9.514 × 10^−6^
F29	4.931 × 10^−4^	1.170 × 10^−9^	2.610 × 10^−10^	4.077 × 10^−11^	6.066 × 10^−11^	8.891 × 10^−10^	3.820 × 10^−10^	8.190 × 10^−1^	1.453 × 10^−1^
F30	3.081 × 10^−8^	8.840 × 10^−7^	4.993 × 10^−9^	1.221 × 10^−2^	3.126 × 10^−11^	8.101 × 10^−10^	4.200 × 10^−10^	3.520 × 10^−7^	3.340 × 10^−11^

**Table A3:** Statistical *p*-values for 9 algorithms in the CEC-2017 (D = 50) evaluation.

Function/ Algorithm	GWO	WOA	COA	IAGWO	DBO	RIME	SO	CPO	SPBO
F1	8.346 × 10^−7^	5.234 × 10^−2^	1.782 × 10^−6^	8.163 × 10^−5^	4.080 × 10^−11^	1.460 × 10^−9^	6.356 × 10^−10^	3.255 × 10^−1^	3.690 × 10^−11^
F2	5.189 × 10^−7^	8.523 × 10^−5^	4.562 × 10^−10^	4.257 × 10^−6^	1.329 × 10^−11^	9.063 × 10^−10^	3.690 × 10^−10^	2.200 × 10^−7^	4.504 × 10^−11^
F3	6.789 × 10^−7^	9.007 × 10^−5^	4.321 × 10^−10^	7.291 × 10^−4^	8.150 × 10^−6^	4.616 × 10^−11^	8.150 × 10^−11^	7.700 × 10^−11^	8.364 × 10^−1^
F4	2.369 × 10^−2^	9.234 × 10^−7^	5.678 × 10^−10^	2.485 × 10^−5^	9.920 × 10^−10^	3.126 × 10^−11^	1.090 × 10^−4^	1.472 × 10^−7^	2.196 × 10^−7^
F5	2.847 × 10^−6^	9.742 × 10^−6^	6.284 × 10^−10^	6.284 × 10^−6^	4.504 × 10^−11^	2.610 × 10^−5^	4.993 × 10^−8^	3.200 × 10^−11^	3.690 × 10^−11^
F6	8.923 × 10^−5^	4.562 × 10^−7^	1.478 × 10^−9^	8.364 × 10^−4^	6.237 × 10^−2^	3.016 × 10^−7^	9.830 × 10^−8^	6.696 × 10^−9^	8.352 × 10^−8^
F7	8.765 × 10^−7^	3.456 × 10^−7^	7.891 × 10^−10^	5.738 × 10^−5^	1.102 × 10^−10^	4.352 × 10^−10^	9.111 × 10^−2^	3.197 × 10^−8^	1.359 × 10^−7^
F8	9.533 × 10^−7^	1.171 × 10^−2^	5.186 × 10^−7^	9.919 × 10^−11^	3.690 × 10^−11^	6.356 × 10^−5^	1.729 × 10^−7^	4.353 × 10^−5^	1.087 × 10^−1^
F9	2.372 × 10^−10^	8.485 × 10^−9^	7.773 × 10^−9^	2.670 × 10^−9^	5.967 × 10^−9^	4.504 × 10^−11^	2.872 × 10^−10^	5.322 × 10^−3^	1.411 × 10^−9^
F10	1.681 × 10^−4^	3.865 × 10^−11^	2.891 × 10^−3^	2.838 × 10^−1^	2.681 × 10^−4^	6.526 × 10^−7^	1.091 × 10^−5^	7.380 × 10^−10^	8.564 × 10^−4^
F11	9.830 × 10^−8^	1.329 × 10^−10^	2.154 × 10^−10^	3.643 × 10^−8^	4.616 × 10^−10^	1.329 × 10^−10^	3.338 × 10^−11^	7.700 × 10^−8^	1.460 × 10^−10^
F12	2.920 × 10^−9^	2.369 × 10^−10^	3.557 × 10^−6^	3.338 × 10^−11^	3.016 × 10^−11^	1.108 × 10^−6^	6.356 × 10^−5^	4.200 × 10^−10^	1.023 × 10^−1^
F13	6.700 × 10^−11^	8.891 × 10^−10^	6.722 × 10^−10^	6.356 × 10^−5^	8.150 × 10^−11^	2.610 × 10^−10^	3.690 × 10^−11^	4.080 × 10^−11^	2.200 × 10^−7^
F14	1.090 × 10^−5^	1.000 × 10^−3^	1.106 × 10^−6^	3.690 × 10^−11^	9.920 × 10^−11^	3.200 × 10^−9^	9.063 × 10^−8^	7.390 × 10^−11^	1.224 × 10^−1^
F15	5.738 × 10^−8^	4.618 × 10^−5^	2.847 × 10^−3^	1.091 × 10^−5^	7.654 × 10^−11^	1.102 × 10^−8^	4.618 × 10^−5^	4.504 × 10^−11^	3.690 × 10^−11^
F16	8.459 × 10^−9^	9.247 × 10^−4^	7.415 × 10^−6^	1.004 × 10^−3^	6.129 × 10^−11^	3.020 × 10^−11^	9.247 × 10^−4^	4.352 × 10^−8^	4.504 × 10^−11^
F17	3.749 × 10^−11^	5.432 × 10^−6^	8.765 × 10^−4^	1.597 × 10^−3^	6.543 × 10^−11^	2.707 × 10^−1^	5.432 × 10^−6^	4.139 × 10^−2^	4.376 × 10^−1^
F18	5.189 × 10^−7^	8.427 × 10^−6^	6.129 × 10^−10^	3.749 × 10^−6^	8.346 × 10^−11^	2.707 × 10^−6^	1.091 × 10^−5^	1.858 × 10^−10^	8.923 × 10^−2^
F19	5.731 × 10^−8^	8.179 × 10^−7^	4.139 × 10^−10^	9.134 × 10^−4^	9.919 × 10^−11^	8.427 × 10^−10^	7.172 × 10^−11^	4.639 × 10^−11^	8.993 × 10^−11^
F20	8.346 × 10^−7^	3.957 × 10^−2^	5.234 × 10^−10^	4.176 × 10^−5^	2.282 × 10^−11^	1.385 × 10^−9^	4.618 × 10^−8^	3.324 × 10^−11^	8.459 × 10^−1^
F21	2.345 × 10^−2^	9.876 × 10^−7^	4.321 × 10^−10^	7.918 × 10^−6^	8.179 × 10^−11^	7.695 × 10^−8^	3.197 × 10^−9^	4.686 × 10^−10^	3.338 × 10^−11^
F22	9.247 × 10^−7^	7.482 × 10^−7^	5.129 × 10^−2^	2.847 × 10^−4^	4.321 × 10^−11^	7.327 × 10^−2^	9.234 × 10^−8^	2.278 × 10^−9^	5.836 × 10^−1^
F23	2.198 × 10^−2^	8.923 × 10^−7^	5.678 × 10^−10^	6.285 × 10^−5^	6.543 × 10^−11^	4.573 × 10^−5^	5.432 × 10^−8^	4.200 × 10^−10^	7.845 × 10^−1^
F24	8.364 × 10^−9^	8.179 × 10^−5^	9.134 × 10^−7^	9.919 × 10^−11^	4.321 × 10^−11^	3.265 × 10^−2^	8.179 × 10^−5^	6.284 × 10^−10^	3.690 × 10^−11^
F25	9.347 × 10^−11^	5.592 × 10^−1^	6.953 × 10^−8^	3.197 × 10^−9^	8.346 × 10^−11^	7.172 × 10^−1^	9.234 × 10^−6^	1.858 × 10^−1^	8.352 × 10^−8^
F26	6.829 × 10^−6^	2.345 × 10^−6^	4.321 × 10^−4^	2.610 × 10^−10^	3.289 × 10^−11^	4.573 × 10^−9^	2.345 × 10^−6^	1.087 × 10^−1^	1.359 × 10^−7^
F27	2.634 × 10^−3^	9.247 × 10^−5^	2.671 × 10^−6^	7.389 × 10^−11^	8.179 × 10^−11^	2.390 × 10^−8^	9.247 × 10^−5^	3.957 × 10^−5^	8.650 × 10^−1^
F28	5.836 × 10^−7^	7.415 × 10^−6^	1.593 × 10^−4^	7.380 × 10^−10^	9.876 × 10^−11^	4.639 × 10^−5^	7.415 × 10^−6^	4.618 × 10^−8^	8.993 × 10^−11^
F29	1.494 × 10^−1^	6.129 × 10^−5^	8.427 × 10^−7^	6.765 × 10^−5^	2.282 × 10^−1^	1.385 × 10^−6^	6.129 × 10^−5^	2.369 × 10^−4^	2.195 × 10^−8^
F30	6.285 × 10^−8^	1.234 × 10^−6^	5.432 × 10^−5^	2.416 × 10^−2^	8.346 × 10^−3^	3.690 × 10^−11^	1.234 × 10^−6^	8.346 × 10^−7^	3.338 × 10^−11^

**Table A4:** Statistical *p*-values for 9 algorithms in the CEC-2017 (D = 100) evaluation.

Function/ Algorithm	GWO	WOA	COA	IAGWO	DBO	RIME	SO	CPO	SPBO
F1	2.153 × 10^−8^	5.280 × 10^−10^	9.637 × 10^−10^	4.840 × 10^−8^	1.274 × 10^−10^	5.860 × 10^−10^	1.672 × 10^−11^	4.630 × 10^−8^	4.504 × 10^−10^
F2	6.283 × 10^−9^	1.880 × 10^−10^	8.354 × 10^−6^	9.470 × 10^−11^	8.736 × 10^−11^	3.870 × 10^−6^	9.772 × 10^−5^	5.430 × 10^−10^	8.352 × 10^−1^
F3	3.340 × 10^−11^	5.860 × 10^−10^	6.143 × 10^−10^	7.420 × 10^−5^	9.637 × 10^−2^	8.450 × 10^−10^	7.528 × 10^−11^	5.280 × 10^−11^	3.689 × 10^−7^
F4	8.101 × 10^−5^	8.930 × 10^−3^	2.917 × 10^−6^	6.280 × 10^−11^	2.483 × 10^−10^	4.740 × 10^−9^	1.846 × 10^−7^	9.760 × 10^−11^	7.844 × 10^−8^
F5	4.352 × 10^−7^	7.160 × 10^−9^	8.736 × 10^−11^	3.730 × 10^−4^	7.492 × 10^−11^	8.290 × 10^−2^	3.958 × 10^−10^	7.160 × 10^−1^	6.567 × 10^−1^
F6	6.129 × 10^−10^	1.580 × 10^−8^	5.826 × 10^−9^	9.640 × 10^−3^	6.143 × 10^−11^	9.470 × 10^−10^	7.418 × 10^−10^	6.930 × 10^−7^	8.534 × 10^−7^
F7	8.427 × 10^−2^	8.450 × 10^−9^	1.274 × 10^−9^	7.530 × 10^−6^	2.917 × 10^−6^	9.720 × 10^−11^	9.677 × 10^−11^	8.290 × 10^−11^	9.514 × 10^−1^
F8	4.980 × 10^−11^	3.753 × 10^−11^	4.800 × 10^−7^	4.200 × 10^−1^	1.110 × 10^−6^	9.470 × 10^−3^	1.500 × 10^−2^	6.360 × 10^−5^	1.110 × 10^−4^
F9	3.324 × 10^−6^	1.698 × 10^−8^	3.474 × 10^−10^	1.464 × 10^−10^	5.573 × 10^−10^	8.650 × 10^−1^	2.610 × 10^−10^	1.019 × 10^−5^	2.090 × 10^−1^
F10	1.206 × 10^−10^	4.353 × 10^−5^	3.871 × 10^−1^	2.052 × 10^−3^	3.352 × 10^−8^	1.868 × 10^−5^	7.394 × 10^−1^	8.993 × 10^−11^	8.120 × 10^−4^
F11	7.283 × 10^−4^	2.923 × 10^−9^	8.445 × 10^−10^	7.188 × 10^−11^	4.629 × 10^−11^	9.776 × 10^−10^	5.861 × 10^−10^	3.877 × 10^−1^	6.722 × 10^−1^
F12	4.200 × 10^−8^	1.581 × 10^−6^	6.927 × 10^−9^	2.757 × 10^−2^	8.354 × 10^−11^	7.425 × 10^−10^	4.925 × 10^−10^	2.646 × 10^−7^	4.440 × 10^−11^
F13	9.760 × 10^−8^	7.164 × 10^−9^	8.830 × 10^−2^	5.436 × 10^−6^	9.637 × 10^−6^	5.287 × 10^−11^	7.492 × 10^−11^	8.368 × 10^−11^	8.190 × 10^−5^
F14	1.030 × 10^−11^	5.835 × 10^−11^	2.174 × 10^−7^	3.965 × 10^−10^	1.274 × 10^−9^	8.643 × 10^−11^	2.385 × 10^−4^	1.854 × 10^−7^	4.077 × 10^−11^
F15	3.100 × 10^−9^	2.487 × 10^−10^	1.672 × 10^−10^	8.938 × 10^−4^	6.284 × 10^−11^	4.745 × 10^−5^	5.826 × 10^−2^	3.963 × 10^−11^	7.283 × 10^−1^
F16	1.210 × 10^−6^	6.845 × 10^−9^	4.183 × 10^−7^	5.933 × 10^−8^	2.846 × 10^−9^	3.736 × 10^−7^	8.294 × 10^−8^	7.161 × 10^−9^	6.356 × 10^−4^
F17	7.220 × 10^−6^	4.056 × 10^−11^	1.110 × 10^−6^	1.780 × 10^−10^	8.890 × 10^−10^	4.920 × 10^−1^	1.490 × 10^−4^	1.220 × 10^−2^	4.440 × 10^−7^
F18	3.500 × 10^−9^	3.340 × 10^−11^	3.160 × 10^−10^	3.245 × 10^−11^	3.730 × 10^−7^	3.080 × 10^−11^	2.020 × 10^−8^	6.280 × 10^−6^	8.500 × 10^−6^
F19	6.070 × 10^−11^	3.964 × 10^−11^	9.260 × 10^−9^	3.320 × 10^−6^	2.150 × 10^−10^	1.070 × 10^−7^	1.860 × 10^−9^	3.470 × 10^−10^	1.330 × 10^−2^
F20	1.070 × 10^−7^	3.470 × 10^−10^	3.110 × 10^−9^	2.230 × 10^−9^	1.470 × 10^−7^	3.010 × 10^−7^	2.760 × 10^−3^	4.930 × 10^−4^	7.290 × 10^−3^
F21	3.990 × 10^−4^	6.070 × 10^−11^	1.330 × 10^−10^	1.370 × 10^−1^	2.150 × 10^−10^	7.220 × 10^−6^	7.700 × 10^−4^	3.080 × 10^−8^	4.220 × 10^−4^
F22	1.610 × 10^−10^	3.470 × 10^−10^	6.530 × 10^−8^	4.640 × 10^−3^	4.570 × 10^−9^	8.290 × 10^−6^	3.590 × 10^−5^	1.610 × 10^−10^	1.870 × 10^−11^
F23	4.080 × 10^−5^	1.190 × 10^−6^	8.890 × 10^−10^	1.890 × 10^−4^	6.720 × 10^−10^	8.680 × 10^−3^	3.780 × 10^−2^	3.340 × 10^−11^	3.040 × 10^−9^
F24	2.847 × 10^−7^	3.456 × 10^−7^	2.369 × 10^−2^	4.562 × 10^−7^	4.139 × 10^−7^	6.129 × 10^−6^	2.345 × 10^−7^	1.220 × 10^−2^	4.440 × 10^−7^
F25	8.170 × 10^−9^	2.859 × 10^−1^	4.130 × 10^−10^	8.326 × 10^−4^	2.977 × 10^−11^	6.124 × 10^−5^	6.143 × 10^−8^	4.918 × 10^−11^	8.650 × 10^−6^
F26	2.870 × 10^−6^	5.886 × 10^−9^	3.720 × 10^−7^	5.846 × 10^−8^	3.726 × 10^−9^	4.356 × 10^−7^	5.861 × 10^−8^	3.730 × 10^−9^	7.890 × 10^−2^
F27	9.140 × 10^−8^	3.793 × 10^−10^	4.620 × 10^−10^	5.826 × 10^−2^	7.418 × 10^−10^	3.555 × 10^−10^	6.839 × 10^−11^	9.479 × 10^−8^	2.820 × 10^−10^
F28	3.460 × 10^−9^	5.823 × 10^−10^	6.130 × 10^−6^	6.144 × 10^−11^	6.143 × 10^−11^	2.143 × 10^−6^	8.445 × 10^−5^	7.426 × 10^−10^	2.570 × 10^−1^
F29	7.910 × 10^−11^	9.737 × 10^−10^	9.430 × 10^−10^	8.475 × 10^−5^	1.274 × 10^−10^	6.898 × 10^−10^	6.839 × 10^−11^	8.294 × 10^−11^	3.460 × 10^−7^
F30	2.350 × 10^−5^	4.814 × 10^−3^	3.260 × 10^−6^	6.814 × 10^−11^	3.726 × 10^−10^	5.833 × 10^−9^	2.846 × 10^−7^	1.227 × 10^−10^	4.610 × 10^−1^

**Table A5:** Statistical *p*-values for 9 algorithms in the CEC-2020 (D = 20) evaluation.

Function/ Algorithm	GWO	WOA	COA	IAGWO	DBO	RIME	SO	CPO	SPBO
F1	8.993 × 10^−11^	3.020 × 10^−11^	3.690 × 10^−11^	8.841 × 10^−7^	4.975 × 10^−11^	1.174 × 10^−9^	4.311 × 10^−8^	1.492 × 10^−6^	5.265 × 10^−5^
F2	6.066 × 10^−11^	2.416 × 10^−2^	1.857 × 10^−9^	3.020 × 10^−11^	3.020 × 10^−11^	6.972 × 10^−3^	3.020 × 10^−11^	6.722 × 10^−10^	5.265 × 10^−5^
F3	8.534 × 10^−1^	3.020 × 10^−11^	5.967 × 10^−9^	7.773 × 10^−9^	2.154 × 10^−6^	2.052 × 10^−3^	9.919 × 10^−11^	5.971 × 10^−5^	7.978 × 10^−2^
F4	3.182 × 10^−4^	3.690 × 10^−11^	1.174 × 10^−9^	8.993 × 10^−11^	6.066 × 10^−11^	2.034 × 10^−9^	4.975 × 10^−11^	1.430 × 10^−5^	7.245 × 10^−6^
F5	1.236 × 10^−3^	2.002 × 10^−6^	8.650 × 10^−4^	1.495 × 10^−1^	2.282 × 10^−1^	1.385 × 10^−6^	2.879 × 10^−6^	9.211 × 10^−5^	1.011 × 10^−8^
F6	5.573 × 10^−10^	1.957 × 10^−10^	1.957 × 10^−10^	5.573 × 10^−10^	5.573 × 10^−10^	1.174 × 10^−9^	1.329 × 10^−10^	1.157 × 10^−7^	5.573 × 10^−10^
F7	6.736 × 10^−6^	3.020 × 10^−11^	8.153 × 10^−11^	3.338 × 10^−11^	1.206 × 10^−10^	2.015 × 10^−8^	1.613 × 10^−10^	1.236 × 10^−3^	2.377 × 10^−7^
F8	3.368 × 10^−4^	4.975 × 10^−11^	1.329 × 10^−10^	6.696 × 10^−11^	3.081 × 10^−8^	5.592 × 10^−7^	4.183 × 10^−9^	1.407 × 10^−4^	4.856 × 10^−3^
F9	1.067 × 10^−7^	1.767 × 10^−3^	5.322 × 10^−3^	9.031 × 10^−4^	4.975 × 10^−11^	7.695 × 10^−8^	6.567 × 10^−2^	4.975 × 10^−11^	1.106 × 10^−4^
F10	1.585 × 10^−4^	5.462 × 10^−9^	8.663 × 10^−5^	2.670 × 10^−9^	1.091 × 10^−5^	3.848 × 10^−3^	7.295 × 10^−4^	1.858 × 10^−1^	6.097 × 10^−3^
